# Hyperactivation of human acidic chitinase (Chia) for potential medical use

**DOI:** 10.1016/j.jbc.2024.108100

**Published:** 2024-12-18

**Authors:** Kazuaki Okawa, Masashi Kijima, Mana Ishii, Nanako Maeda, Yudai Yasumura, Masayoshi Sakaguchi, Masahiro Kimura, Maiko Uehara, Eri Tabata, Peter O. Bauer, Fumitaka Oyama

**Affiliations:** 1 Department of Chemistry and Life Science, Kogakuin University, Hachioji, Tokyo, Japan; 2 School of Bioscience and Biotechnology, Tokyo University of Technology, Hachioji, Tokyo, Japan; 3 Japan Society for the Promotion of Science (PD), Tokyo, Japan; 4 Bioinova a.s., Prague, Czech Republic

**Keywords:** acidic chitinase (Chia), amino acid substitutions, chitin, enzyme engineering, evolution, exon swapping, hyperactivation, primate lineage, treating pulmonary diseases

## Abstract

Accumulation of environmental chitin in the lungs can lead to pulmonary fibrosis, characterized by inflammatory infiltration and fibrosis in acidic chitinase (Chia)-deficient mice. Transgenic expression of Chia in these mice ameliorated the symptoms, indicating the potential of enzyme supplementation as a promising therapeutic strategy for related lung diseases. This study focuses on utilizing hyperactivated human Chia, which exhibits low activity. We achieved significant activation of human Chia by incorporating nine amino acids derived from the crab-eating monkey (*Macaca fascicularis*) Chia, known for its robust chitin-degrading activity. The modified human Chia retained high activity across a broad pH spectrum and exhibited enhanced thermal stability. The amino acid substitutions associated with hyperactivation of human Chia activity occurred species specifically in monkey Chia. This discovery highlights the potential of hyperactivated Chia in treating pulmonary diseases resulting from chitin accumulation in human lungs.

Chitin, a polymer of *N*-acetyl-D-glucosamine linked by β-1, 4-bonds, serves as a fundamental structural component in the exoskeleton of crustaceans and insects, in the microfilaria sheath of parasitic nematodes and the cell walls of fungi (1, 2). Chitinases are enzymes that hydrolyze the β-1, 4 glycosidic bonds within the chitin polymer (1, 2, 3).

Acidic chitinase (Chia), also known as acidic mammalian chitinase AMCase (4), encoded by the *Chia* gene, has been associated with inflammatory lung diseases (5, 6) and plays a crucial role in the breakdown of chitin. The expression and activity levels of Chia are significantly altered in various diseases such as asthma and allergic inflammation (7, 8, 9).

The accumulation of chitin from environmental sources such as mites and molds, associated with low activity of Chia in Chia-deficient mice, leads to chitin buildup in the airways. This buildup was mitigated by transgenically expressed Chia, reducing sustained inflammatory pathways associated with lung diseases and alleviating related symptoms (10, 11). These findings indicate the potential impact of chitinase activity on pulmonary conditions like asthma and fibrosis in humans, where active Chia could alleviate these diseases (11, 12).

The ancestors of placental mammals emerged as small insect-eating organisms that evolved shortly after the extinction of dinosaurs (13). Chia has been identified as a molecular record of the evolutionary process of the ancestors of insect-eating mammals (14). Chia enzymes in insect-eating and omnivorous animals such as mice, pigs, marmosets, and crab-eating monkeys have preserved complete ORFs and exhibit high chitinolytic activity (15, 16, 17, 18, 19). Conversely, in noninsect-eating carnivorous species, Chia has undergone inactivation or pseudogenization (20). In herbivorous animals, Chia has undergone genetic alterations, leading to a molecule with diminished activity due to the absence of chitin in their diet (21), highlighting a clear correlation between a chitin-containing diet and Chia functionality (20, 21, 22).

Compared to mouse Chia, the human enzyme exhibits significantly lower chitinase activity, with wild-type human Chia (WT Chia) having its peak activity at pH 5.0 and approximately 50 times lower activity (9, 23, 24, 25). However, previous research has shown that substituting R61 with methionine in human Chia shifts its optimum pH to 2.0, resulting in a level of activation comparable to that in mice (25). Chia activity in other species, such as dogs and cattle, can also be enhanced through specific amino acid substitutions (20, 21). Our strategy for enzyme activation combines biochemical and evolutionary approaches, aiming to create highly active Chia variants for therapeutic applications (11, 12).

Crab-eating monkeys (*Macaca fascicularis*) are used as a nonhuman primate model for biomedical research in this field, given their diet comprising chitin-containing organisms such as crabs, crustaceans, and insects (26, 27, 28). Monkey Chia exhibits optimal activity at pH 5.0, maintaining its functionality within a broad pH range and at elevated temperatures (19, 29, 30, 31). Moreover, monkey Chia is 16 times more potent than mouse Chia at pH 5.0 (19), offering insights into structural modifications to enhance human Chia activity for therapeutic purposes.

In this study, we first replaced specific regions of human Chia with monkey sequences. Among the generated chimeric human/monkey Chia enzymes, some of these proteins exhibited activity levels surpassing those of the monkey enzyme. Subsequent investigations identified nine amino acids in monkey Chia that can activate human enzymes. These findings indicate the compelling potential of utilizing hyperactivated Chia in treating lung diseases arising from chitin accumulation.

## Results

### Identification of a highly active human/monkey Chia chimera

**Figure 1:**
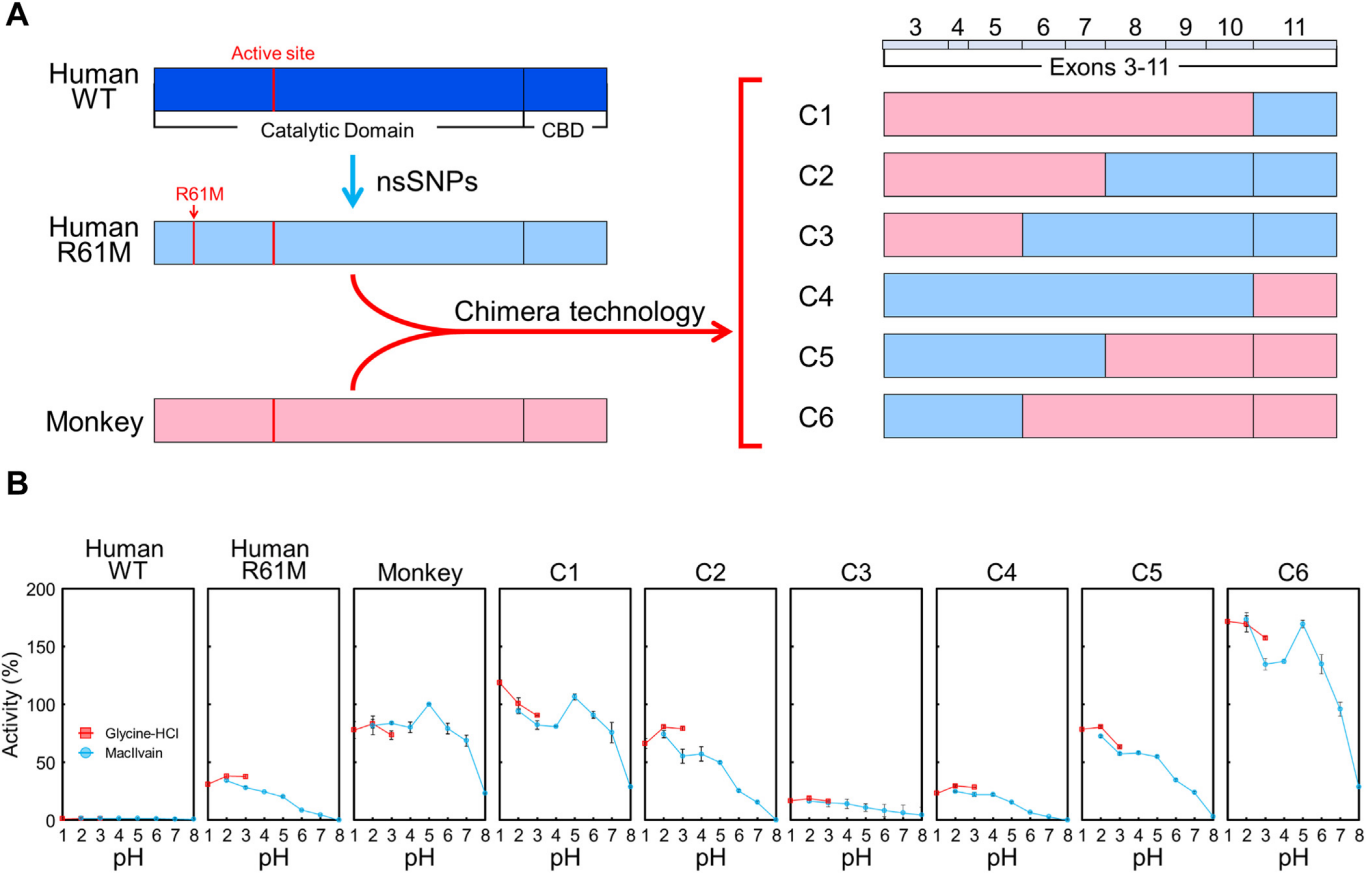
**Hyperactivation of human Chia by substituting multiple exons from monkey Chia.***A*, schematic representation of *Escherichia coli*–expressed human WT and R61M Chia, monkey Chia, and chimeric Chia fusion proteins (C1–C6). Amino acid sequences are color-coded: *pink* for monkey Chia and *blue* for human R61M Chia. *B*, chitinolytic activities of human WT and R61M Chia, monkey Chia, and chimeric Chia fusion proteins (C1–C6). Monkey Chia activity at pH 5.0 was set as 100% and depicted as relative activity. Error bars represent the mean ± SD from a triplicate experiment. Chia, acidic chitinase.

For chimeric protein engineering, we used human Chia with the R61M substitution (designated as R61M Chia), a mutant displaying approximately 50-fold higher activity than the WT Chia (25). R61M Chia was combined with crab-eating monkey Chia (monkey Chia) (Fig. 1*A*), and the created chimeras were then expressed. Their activity was assessed using the synthetic fluorogenic substrate 4-methyl umbelliferyl β-D-*N*, *N′*-diacetyl chitobioside [4-MU-(GlcNAc)_2_].

In chimeras C1 to C3, where monkey Chia was progressively substituted with human Chia from the C terminus, chitinase activity showed a negative correlation with the portion of the human sequence (Fig. 1*B*). Conversely, in C4 to C6, where human Chia was incrementally substituted with monkey Chia from the C terminus, chitinase activity displayed a positive correlation with the length of the monkey sequence.

Notably, chimera C6, comprising human exons 3 to 5 and monkey exons 6 to 11, exhibited significantly higher chitinase activity than any other tested molecule, including monkey Chia, across a broad pH range (pH 1.0–7.0) (Fig. 1*B*). These results suggest that the activation of human Chia depends on contributions from one or more of the monkey Chia exons 6 to 11.

### Identification of exons and amino acids required for hyperactivation of human Chia

**Figure 2:**
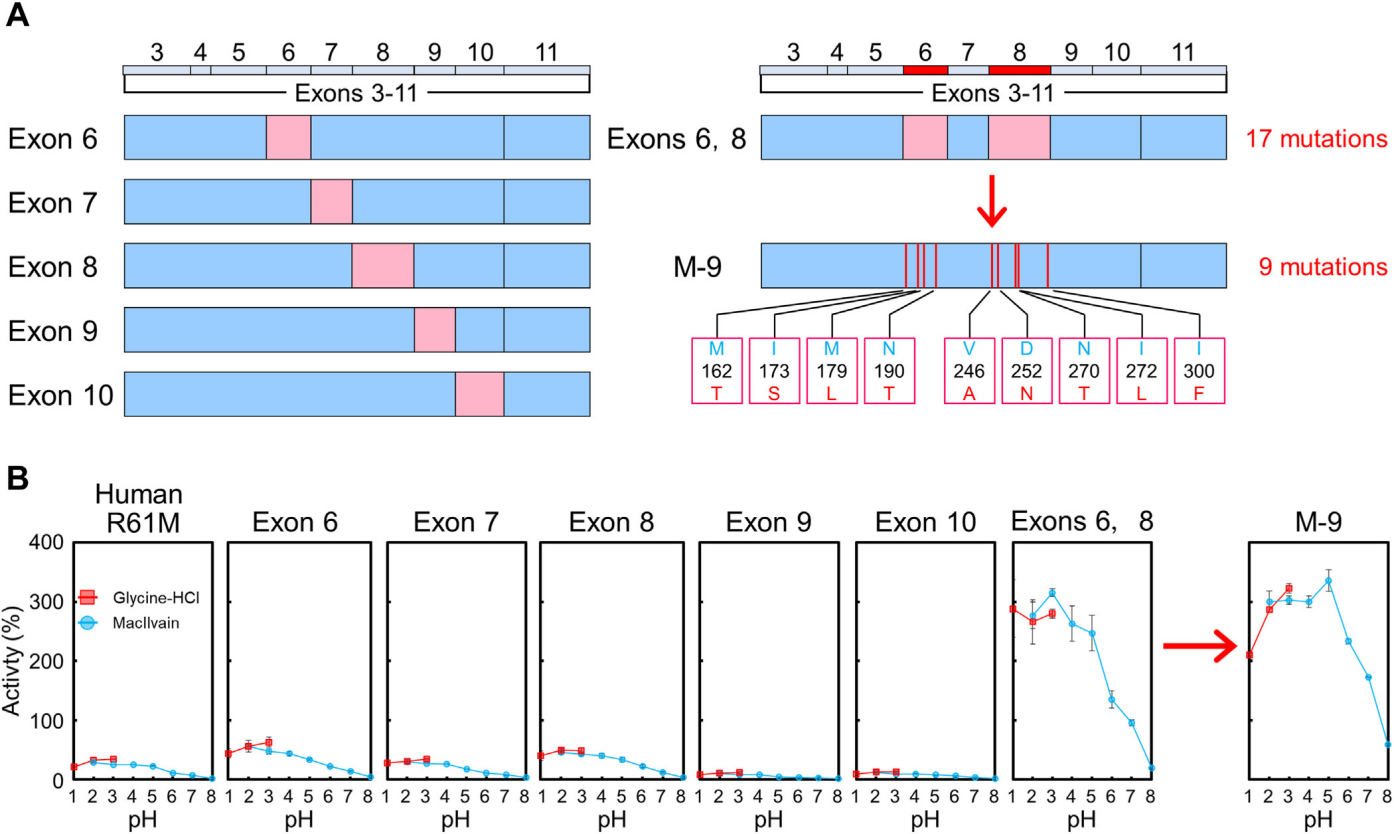
**Hyperactivation of human Chia by substituting nine amino acids from monkey Chia.***A*, schematic representation of the Chia chimeric protein. *Left*: Chia chimera with monkey-derived amino acid sequences substituted in exons 3 to 11 of the Chia coding region. *Right*: A mutant in which monkey-derived amino acid substitutions were introduced into exons 6 and 8 of human Chia. Amino acids from human or monkey Chia are shown in *blue* and *pink*, respectively. *B*, chitinolytic activities of Chia chimeric proteins. Monkey Chia activity at pH 5.0 was set as 100% and shown as relative activity. Error bars represent the mean ± SD from a triplicate experiment. Chia, acidic chitinase.

We constructed several additional chimeras to pinpoint the regions responsible for the enzyme hyperactivation observed in chimera C6. Surprisingly, replacing human exons 6 to 10 with the corresponding monkey sequences did not result in significant activation (Figs. 2*A*, left, and 2*B*). This finding was unexpected, as it indicates that the hyperactivation of human Chia requires more complex interactions spanning multiple exons.

Consequently, multiple exon substitutions were performed (Figs. 2*A*, right and S1*A*). Strikingly, we found that only the simultaneous replacement of exons 6 and 8 resulted in a hyperactive enzyme (Figs. 2*B* and S1*B*).

Next, we investigated which amino acids differing between human and monkey sequences in these two exons are responsible for the observed activity differences. Among 17 amino acid variations (Fig. 2*A*, right), we identified nine residues necessary for the activation of human Chia (referred to as M-9, mutant with nine amino acids) (Figs. 2*B* and S2–S4). M-9 Chia includes four amino acid substitutions in exon 6 (M162T, I173S, M179L, N190T) and five amino acid substitutions in exon 8 (V246A, D252N, N270T, I272L, and I300F) (Figs. 2*A*, right and 2*B*).

### Enzymatic properties of M-9 Chia at different pH and temperatures

**Figure 3:**
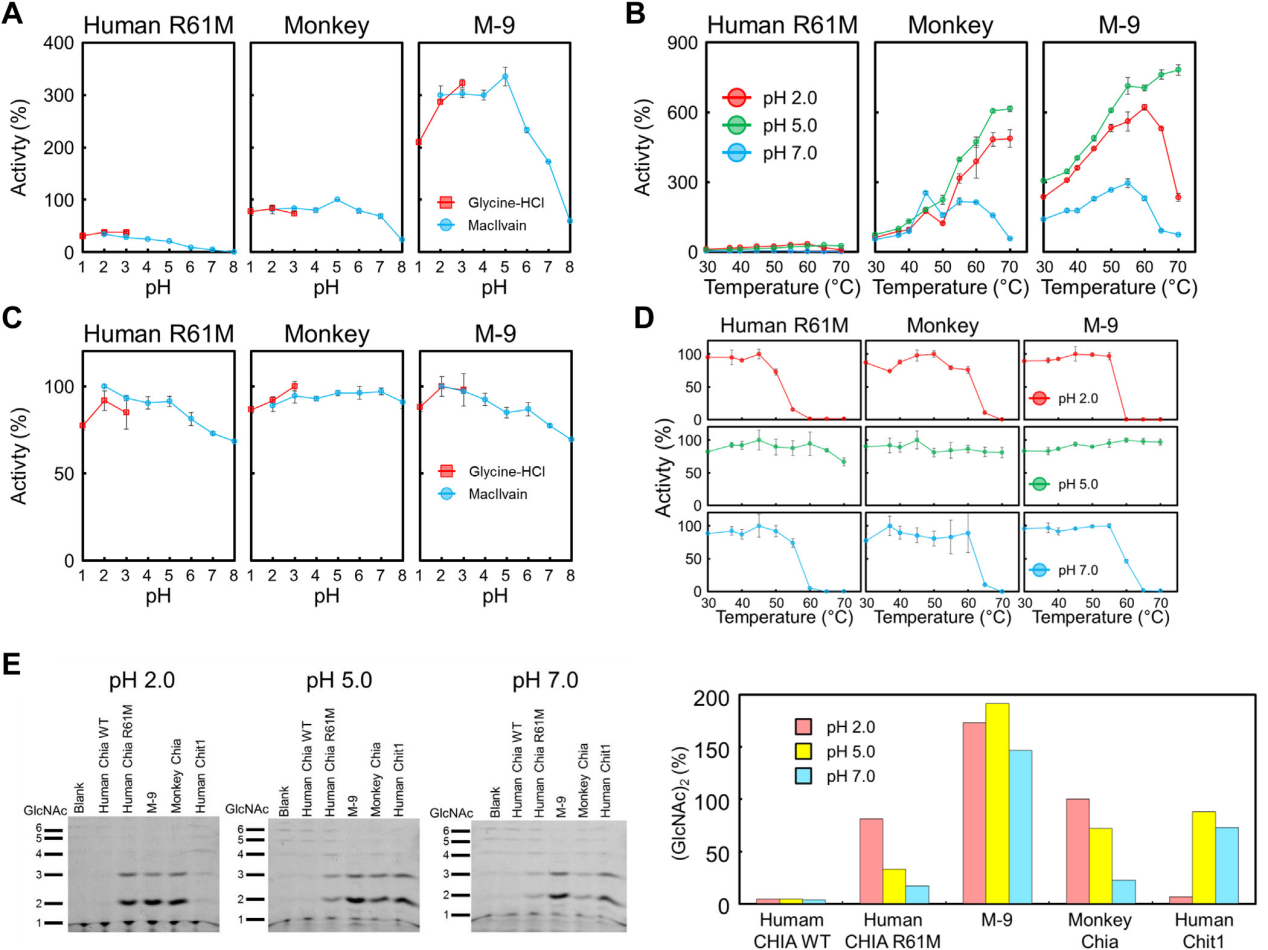
**Enzymatic properties of M-9, a hyperactivated human Chia mutant, under various conditions.***A*, optimal pH for M-9 compared with monkey and human Chia R61M. Chitinolytic activities were measured at pH 1.0 to pH 8.0. Monkey Chia activity at pH 5.0 was set as 100% and shown as relative activity. Values represent the average of three measurements. Error bars indicate the mean ± SD from a triplicate experiment. *B*, temperature dependence of M-9 compared with monkey and human Chia R61M. Temperature profiles from 30 °C to 70 °C for each Chia. Monkey Chia activity was set as 100% and shown as a relative activity. Reaction conditions are color-coded: *red* for pH 2.0, *green* for pH 5.0, *blue* for pH 7.0. *C*, pH stability of M-9 and parental Chia proteins at 37 °C. The stability of each Chia protein was assessed by pre-incubating them at different pH levels (ranging from pH 1.0 to pH 8.0) for a specified duration. The chitinase activity of each Chia protein after preincubation was measured and expressed as a percentage relative to its maximum activity under optimal conditions. Monkey Chia activity at its optimal pH was set as 100%. Error bars represent the mean ± SD from triplicate experiments. *D*, thermal stability from 30 °C to 70 °C for each Chia. The maximum activity of each Chia was set as 100% and shown as relative activity. Reaction conditions are color-coded: *green* for pH 5.0 and *blue* for pH 7.0. *E*, chitin degradation activity of hyperactivated Chia for high-molecular-weight chitin. *Left*: comparison of chitinase activity of each mammalian chitinase on colloidal chitin under pH 2.0, 5.0, and 7.0 conditions. Degradation products were analyzed using the FACE method. *Right*: quantification data for (GlcNAc)_2_. Reaction conditions are color-coded: *pink* for pH 2.0, *yellow* for pH 5.0,and *blue* for pH 7.0. Values are shown relative to the maximum amount of monkey Chia degradation products (pH 2.0) set at 100%. FACE, fluorophore-assisted carbohydrate electrophoresis; Chia, acidic chitinase.

We conducted a comprehensive analysis comparing the enzymatic properties of M-9 Chia with those of the parental monkey and human R61M Chia enzymes. The pH dependence of M-9 was assessed by measuring chitinolytic activity at 37 °C for 5 min across a pH range of 1.0 to 8.0. Notably, M-9 Chia exhibited significantly higher activity than the monkey and human R61M enzymes under all pH conditions, with peaks at pH 5.0 and 2.0 (Fig. 3*A*).

Evaluation of the temperature effect (30–70 °C) on chitinase activity revealed that M-9 Chia displayed increasing activity with higher temperatures, reaching maximum activity at 70 °C, 60 °C, and 55 °C at pH 5.0, 2.0, and 7.0, respectively (Fig. 3*B*). Importantly, M-9 Chia surpassed the activities of the parental enzymes under almost all respective conditions, particularly excelling at temperatures close to human physiological levels.

Subsequent assessment of pH stability (preincubation at pH 1–8 at 37 °C) indicated that M-9 Chia exhibited a stability profile resembling human Chia, maintaining over 80% chitinase activity across the tested pH range (Fig. 3*C*).

Evaluation of the thermostability (at pH 2.0, 5.0, and 7.0) revealed that M-9 Chia remained stable at all temperatures at pH 5.0, similar to monkey Chia. However, at pH 7.0, a decline and absence of activity were observed at 60 °C and ≥65 °C, respectively, consistent with the behavior of human R61M Chia (Fig. 3*D*). These results highlight the excellent enzymatic properties of M-9 Chia endowed by nine monkey Chia amino acid substitutions in the human enzyme.

Lastly, we compared the degradation of high molecular weight colloidal chitin (p-chitin) by M-9 Chia and other chitinases (human WT Chia, R61M Chia, human chitotriosidase (Chit1) (20, 32, 33, 34), and monkey Chia). M-9 Chia exhibited superior chitinase activity for p-chitin compared to human WT Chia, R61M Chia, and human Chit1, as well as monkey Chia, at each tested pH (2.0, 5.0, and 7.0) (Fig. 3*E*). These findings highlight the robust chitinase activity of M-9 Chia toward chitin substrates under physiological conditions, exceeding that of other tested enzymes.

### Essential amino acid substitution for hyperactivation of M-9 Chia

**Figure 4:**
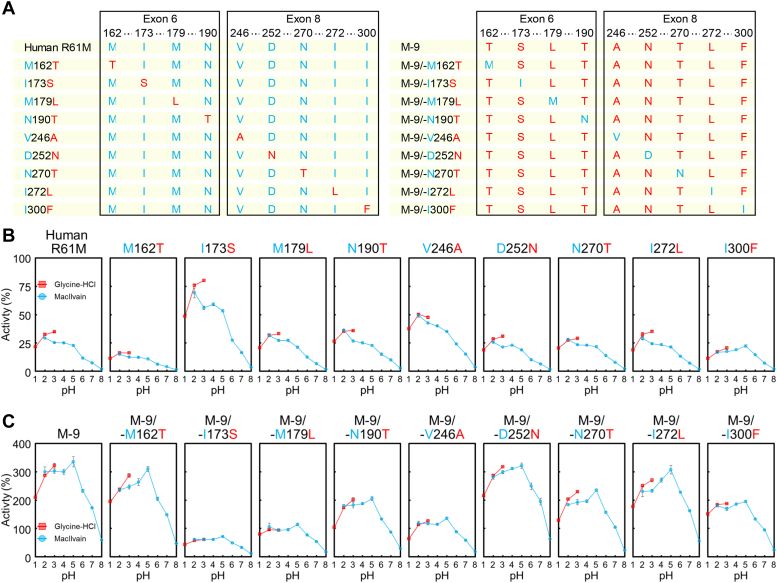
**Identification of critical amino acid substitutions for hyperactivation of human Chia.***A*, sequence diagram illustrating the nine amino acid substitutions involved in hyperactivation. *Left*: human Chia mutant with single amino acid substitutions among the nine residues associated with hyperactivation. *Right*: mutant, where one amino acid residue out of the nine involved in hyperactivation is reverted to the human type. *B*, chitinolytic activities of human R61M Chia and single amino acid substitution human Chia mutants, measured after incubation for 30 min at 37 °C in McIlvaine’s buffer. *C*, chitinolytic activities of M-9 and eight amino acid substitution human Chia mutants, measured under the same conditions. Monkey Chia activity at pH 5.0 was set as 100% and shown as relative activity. Error bars represent mean ± SD from triplicate experiments. Chia, acidic chitinase.

To identify individual amino acid substitutions associated with hyperactivation in M-9 Chia, we generated mutants where only one amino acid substitution out of the nine residues of M-9 Chia was introduced into R61M Chia (Fig. 4*A*, left panel). Introducing I173S or V246A into R61M Chia notably enhanced chitinase activity compared to the parental enzyme (Figs. 4*B* and S5). Furthermore, only substituting I300F in R61M Chia among these mutants resulted in peak activity at pH 5 (Fig. 4*B*). The contrasting optimal pH values between R61M Chia (pH 2.0) and I300F (pH 5.0) suggest a pivotal role of I300F in modulating the optimal pH of M-9 Chia.

Subsequently, we generated mutants where each of the nine amino acid residues in M-9 Chia was replaced with the corresponding residue from human Chia (Fig. 4*A*, right panel). Comparative analysis revealed a significant reduction in chitinase activity in the human Chia mutants without I173S or V246A substitutions compared to M-9 Chia (Fig. 4*C*). These findings highlight the critical importance of I173S and V246A as key amino acid substitutions contributing to the hyperactivation of human Chia.

### Identification of key residues I173S and V246A in M-9 Chia hyperactivation

**Figure 5:**
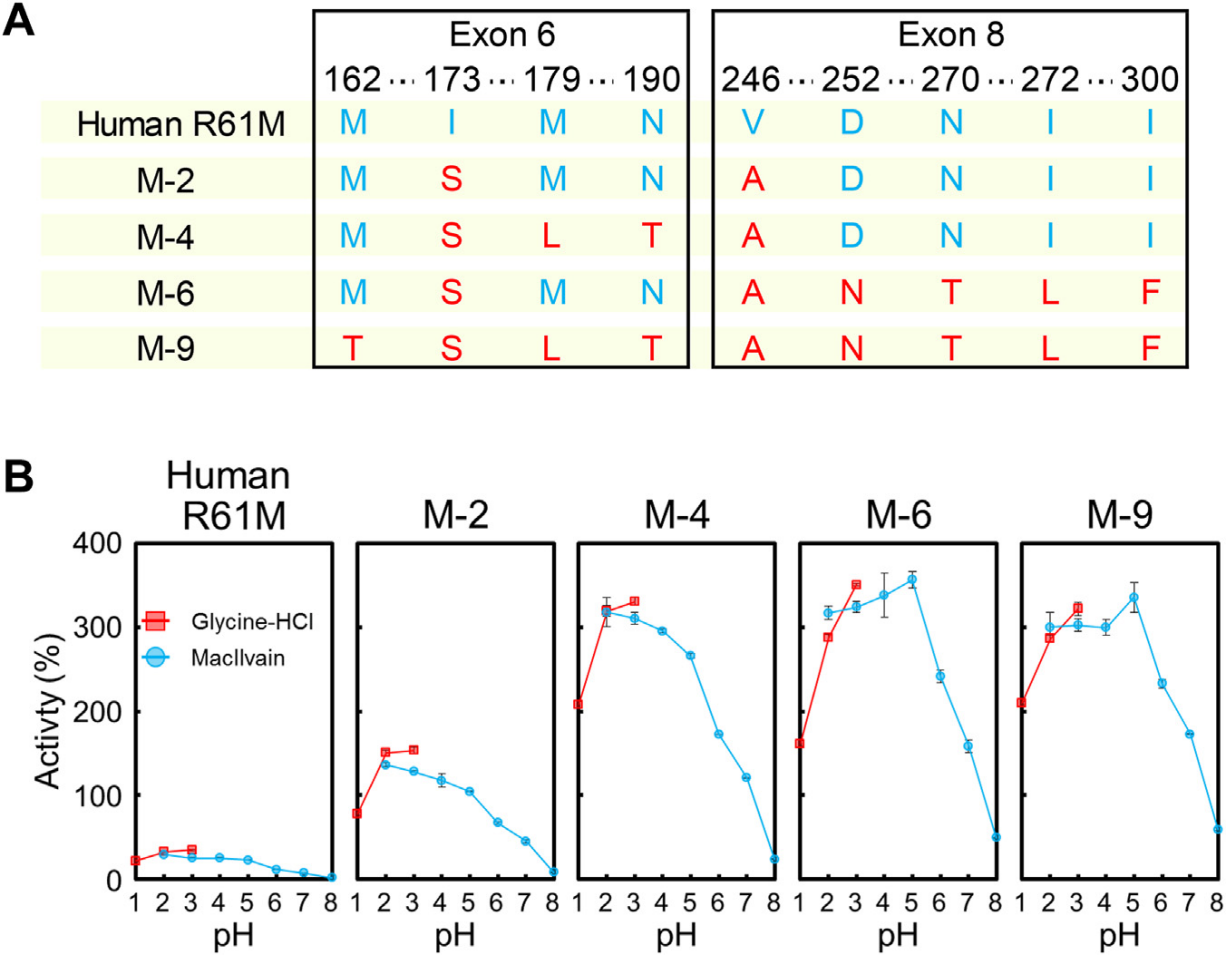
**I173S and V246A are key amino acid substitutions governing the hyperactivation of human Chia.***A*, sequence diagram showing the nine amino acid substitutions involved in hyperactivation of the generated mutant. *B*, chitinolytic activities of human R61M, M-2, M-4, M-6, and M-9 Chia proteins, measured after incubation for 30 min at 37 °C in McIlvaine’s buffer. Monkey Chia activity at pH 5.0 was set as 100% and shown as relative activity. Chia, acidic chitinase.

Upon comparing the chitinase activities of various M-9 Chia mutants, it became apparent that I173S and V246A play the most crucial roles in the hyperactivation of M-9 Chia. To further elucidate their significance, we introduced I173S in exon 6 and V246A in exon 8 into human R61M Chia, obtaining M-2 Chia (Fig. 5*A*). Although M-2 Chia showed increased chitinase activity, its performance fell short of the hyperactivation levels achieved by M-9 Chia (Fig. 5*B*).

We introduced additional amino acid substitutions associated with high activation to explore the underlying mechanisms of exon 6 and exon 8 of M-2 Chia. The resulting mutants, M-4 Chia (I173S, M179L, N190T in exon 6 and V246A in exon 8) and M-6 Chia (I173S in exon 6 and V246A, D252N, N270T, I272L, and I300F in exon 8), exhibited significant hyperactivation. These findings further underscore the pivotal roles of I173S and V246A in driving the hyperactivation observed in M-2, M-6, and M-9 Chia (Figs. 5, *A* and *B* and S6).

### Kinetic evaluation of enhanced catalytic efficiency in Chia mutants

For enzyme kinetic analysis, we compared the rate constants (*k*_cat_ and *K*_m_) of human Chia and its mutants using both synthetic and natural substrates. When synthetic substrates were used, the M-2 mutant (I173S and V246A substitutions) exhibited a marked increase in *k*_cat_ value, indicating an improved catalytic turnover rate (Table 1).

**Table 1:** Kinetic parameters of human Chia and hyperactivated Chia mutants for 4-MU-(GlcNAc)_2_

Chia	*K*_m_ [**μ**M]	*k*_cat_ [min^−1^]	*k*_cat_/*K*_m_ [min^−1^ **μ**M^−1^]
Human R61M	76.19	28.45	0.37
M-2	54.49	128.41	2.36
M-4	82.68	189.83	2.30
M-6	156.00	511.15	3.28
M-9	200.79	750.54	3.74

4-MU-(GlcNAc)_2_, 4-methyl umbelliferyl β-D-*N*, *N′*-diacetyl chitobioside.

Mutants with additional amino acid substitutions beyond those in M-2 demonstrated higher *k*cat/*K*m ratios, signifying enhanced catalytic efficiency than WT and R61M human Chia. Among these, M-6 and M-9 showed the most significant increases in *k*cat, suggesting a notable improvement in catalytic turnover. However, these mutants also exhibited increased *K*_m_ values, reflecting a decreased substrate affinity under synthetic substrate conditions.

In experiments using natural substrates, the *V*_max_/*K*_m_ ratio of M-9 Chia was higher than that of R61M human Chia, confirming its improved catalytic efficiency (Table 2). However, the enhancement observed with M-9 when using natural substrates was less pronounced than that seen with synthetic substrates. Specifically, the *k*_cat_/*K*_m_ of R61M for synthetic substrates was 0.37, while that of M-9 was 3.74 at pH 5.0. In contrast, the *V*_max_/*K*_m_ of R61M for natural substrates was 0.08, whereas that of M-9 was 0.13 at pH 5.0. These results indicate a ∼10-fold improvement in catalytic efficiency for synthetic substrates, but only a 1.6-fold improvement for natural substrates. This disparity suggests that substrate specificity plays a significant role in the observed activity differences between oligomeric and high-molecular-weight substrates.

**Table 2:** Kinetic parameters of human Chia and hyperactivated Chia mutants for p-chitin

Chia	*K*_m_ [mg/ml]	*V*_max_ [min^−1^]	*V*_max_/*K*_m_ [min^−1^ (mg/ml)^−1^]
Human R61M	0.32	0.02	0.08
M-2	1.57	0.04	0.03
M-4	1.15	0.03	0.03
M-6	0.27	0.05	0.19
M-9	0.34	0.05	0.13

These findings suggest that the introduction of multiple amino acid substitutions in M-9 induces substantial structural changes, resulting in enhanced chitinase activity that is less dependent on substrate type. Specifically, the structural modifications involving I173S and V246A are likely to optimize the spatial alignment between the catalytic residues and the substrate, thereby enhancing overall enzymatic performance.

### Evolutionary analysis of chitinase amino acid sequences

**Figure 6:**
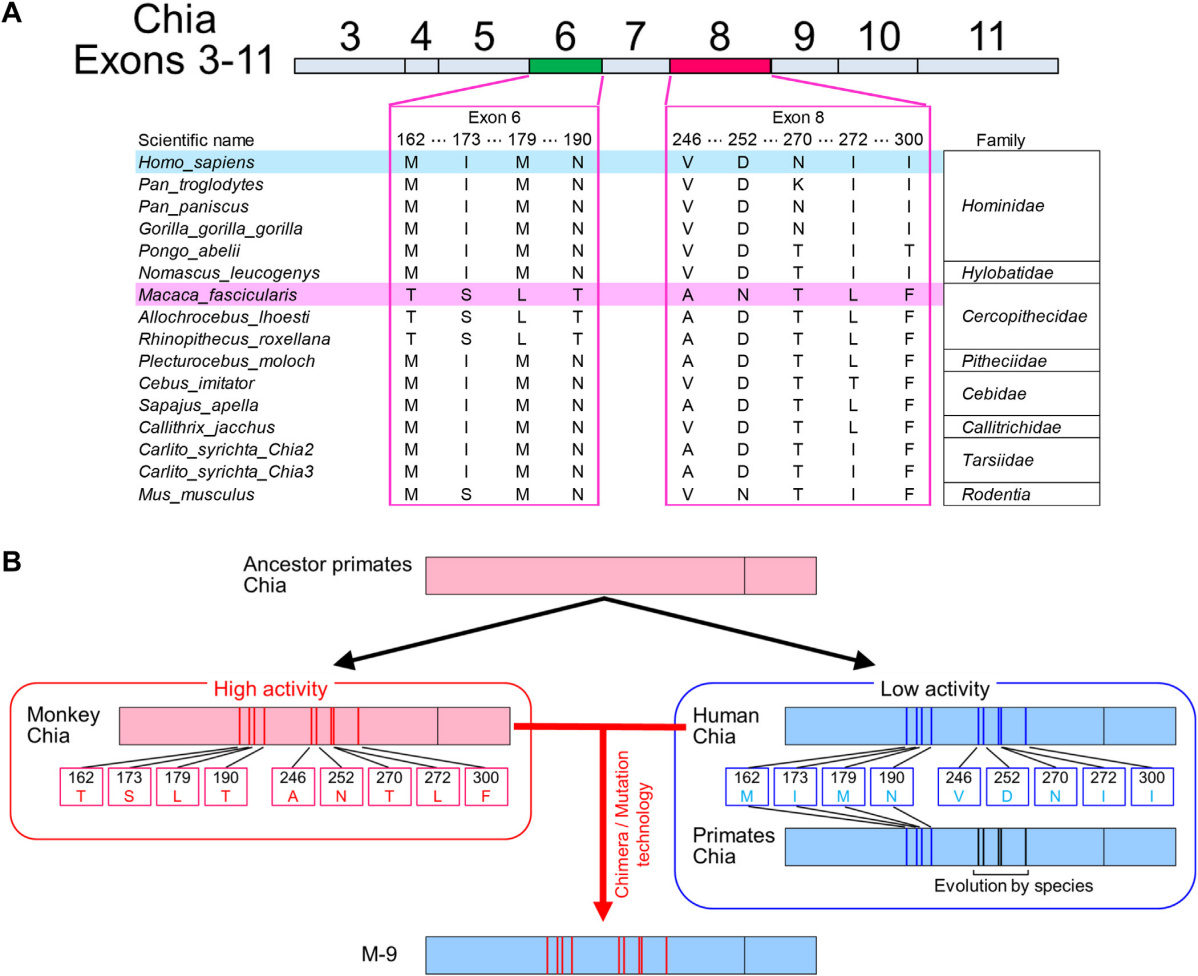
**Hyperactivation of human Chia is attributed to amino acids with high activity in monkey Chia.***A*, sequence diagram comparing the amino acid sequences of exons 6 and 8 of primate Chia proteins. *B*, conceptual diagram illustrating the divergence of ancestral genes of human and monkey Chia into humans and monkeys. Monkey Chia acquired high chitinase activity during the evolutionary process, with the amino acids responsible for it hyperactivating human Chia. Chia, acidic chitinase.

Upon examining the amino acid sequences of Chia across mammalian primates, a notable distinction was observed: all four amino acids in exon 6 were exclusive to the Cercopithecidae family, not found in other primate families (Figs. 6*A*, S7 and S8). This suggests that the mutations observed in Chia exon 6 within the Cercopithecidae family, including *M. fascicularis*, evolved uniquely within this lineage to enhance chitinase activity. In contrast, the five amino acids identified in exon 8 were also present in species beyond Cercopithecidae, indicating their broader evolutionary conservation and distinct origins from the ancestral mammalian Chia sequences.

Notably, while exon 6 of many primate Chia sequences share similarity with human Chia, suggesting reduced activity, crab-eating monkey Chia and its relatives evolved to retain activated Chia by introducing specific mutations in exon 6. These findings confirm that the enhanced chitinase activity observed in crab-eating monkey Chia evolution can be successfully transferred to human Chia, resulting in hyperactivation (Fig. 6*B*).

Based on these findings, we propose that the ancestral Chia enzyme likely originated from a common ancestor shared by placental mammals (Fig. 6*B*). This ancestral enzyme would have exhibited basic chitinase activity, potentially as an adaptive response to the insectivorous dietary habits of early mammals (13, 14). As mammals diversified and adopted varied dietary preferences, evolutionary pressures drove the development of species-specific variations in Chia activity (14, 20, 21, 22, 28). These evolutionary adaptations underscore the dynamic interplay between chitinase genes and their environmental contexts, highlighting their critical roles in host–pathogen interactions and broader environmental adaptations.

## Discussion

Chitin accumulation in the lungs is a hallmark of pulmonary fibrosis, often leading to chronic inflammation and tissue scarring, as observed in Chia-deficient mice (2, 5, 11, 35). Inspired by the robust chitinase activity observed in the crab-eating monkey (*M. fascicularis*) Chia, which exhibits optimal activity at pH 5.0, our study aimed to enhance the catalytic efficiency of human chitinase through protein engineering techniques to explore its potential for therapeutic applications (19, 31). We identified key amino acid substitutions that significantly enhance enzymatic activity by systematically swapping exons between human and monkey Chia and conducting extensive mutagenesis studies.

Our primary focus is the discovery of specific amino acid substitutions that significantly enhance Chia's activity. In this context, Barad *et al.* identified A239T and V246A as key mutations enhancing chitinase activity through an error-prone PCR method, which they describe as an evolutionary experimental approach (12). This method provides a rapid, laboratory-based means of identifying activating mutations. In contrast, our study adopts a long-term evolutionary perspective, selecting nine amino acid substitutions from crab-eating monkey Chia, a primate with chitin-rich diet. This approach reflects the species' biological pressures and environmental contexts over a prolonged evolutionary timescale.

Notably, V246A is one of the nine substitutions in our M-9 variant, underscoring its critical role in chitinase activation. Our findings validate results of Barad *et al.* (12), highlighting V246A as one of the two most pivotal residues (alongside I173S) for achieving the hyperactivation observed in M-9. This consistency across studies emphasizes V246A’s essential role in modulating chitinase activity.

Interestingly, while Barad *et al.* identified A239T as another activity-enhancing mutation, it was not included in our M-9 variant (12). This discrepancy likely reflects differences in evolutionary pressures and substrate specificities between experimental and naturally evolved systems. These findings illustrate the complementary nature of experimental and evolutionary approaches in enzyme optimization, demonstrating how laboratory-based mutagenesis and natural selection insights can synergistically guide the development of highly efficient chitinase variants.

Although experimental framework of Barad *et al.* provides valuable insights into chitinase activity, replicating their method posed practical challenges due to the high cost and limited availability of the enzyme they used (12). Additionally, a conventional approach employing high-molecular-weight substrates proved infeasible in our hands. We adopted a colloidal chitin method to overcome these limitations, analyzed *via* fluorophore-assisted carbohydrate electrophoresis (FACE) (36). This technique allowed us to effectively quantify the degradation of high-molecular-weight chitin (p-chitin) under various conditions. In this report, we extended the application of this method to include comprehensive kinetic studies, calculating parameters such as *k*_cat_, *K*_m,_ *k*_cat_/*K*_m_, and *V*_max_/*K*_m_ for M-9.

Our kinetic analysis revealed a ∼10-fold improvement in catalytic efficiency with synthetic substrates compared to a 1.6-fold improvement with natural substrates. This disparity likely reflects differences in substrate accessibility and enzyme–substrate interaction mechanisms. Synthetic substrates, being smaller and more uniform, likely allow unrestricted access to the active site. In contrast, high-molecular-weight chitin may present steric or conformational barriers due to the interplay between the catalytic domain and the chitin-binding domain of M-9 Chia. Future studies focusing on the isolated catalytic domain of M-9 Chia could elucidate these structural adaptations and provide deeper insights into the mechanisms required for efficient interaction with high-molecular-weight chitin, potentially enhancing its therapeutic applications.

Our analyses demonstrated that the R61M variant substantially enhances chitinase activity compared to WT human Chia (WT Chia), which exhibits minimal activity (Fig. 3*E*) (25). The R61M substitution resulted in an approximately 70-fold increase in activity with synthetic substrates. In this study, we found that the M-9 variant, incorporating nine additional amino acid substitutions, further enhanced the activity of R61M by 10-fold for synthetic substrates and 1.6-fold for natural substrates. Given that WT Chia shows negligible activity on natural chitin, the R61M and M-9 variants represent significant advancements in chitinase engineering for therapeutic applications. These findings highlight the critical role of the R61M substitution in enhancing WT Chia activity and underscore the catalytic efficiency of M-9 in effectively degrading natural chitin deposits.

Our previous study revealed a reversal in the optimal pH of human R61M Chia, shifting from pH 5.0 to 2.0 (25). In this study, we demonstrated that introducing nine amino acid substitutions restored the optimal pH of R61M Chia, bringing it back toward neutral conditions. Recently, Díaz *et al.* provided valuable insights into the dual pH optima of mAMCase (mouse Chia), highlighting the critical role of the DxxDxDxE motif in modulating enzymatic activity across varying pH conditions (37). Their analysis illustrated how specific residues adapt to acidic and neutral environments through changes in protonation states. Building on these findings, we propose that several of the nine substitutions in M-9 influence the catalytic environment, contributing to the observed pH shift.

Olland *et al.* identified key second-shell residues (H208, H269, and R145) in human Chia that modulate the pKa of active site residues and impact structural conformation (38). Bussink *et al.* further emphasized the role of H208 in determining the enzyme’s pH optimum (39). Our study leverages these structural insights, suggesting that substitutions in M-9, particularly V246A and I173S, may similarly alter the conformational dynamics and hydrogen bonding network around the catalytic site. These changes are hypothesized to optimize enzyme–substrate interactions under physiologically relevant pH conditions, aligning with findings of Barad *et al.* on V246A (12). Preliminary data from our ongoing research suggest that substitutions at His208 and H269 in human Chia may also contribute to pH optima shifts, a phenomenon observed in certain avian and mammalian Chia paralogs. Although the nine substitutions in M-9 do not directly affect first-shell catalytic residues, their positions in the second shell (and potentially third shell) are likely to influence enzymatic function indirectly. This supports the notion that structural changes at the second-shell level can fine-tune Chia activity by modulating the active site's chemical environment and substrate affinity.

Future studies, including crystal structure determination of M-9, will be crucial to validate these proposed mechanisms. Such studies will provide a deeper understanding of how these substitutions enhance chitinase activity and pH adaptability. Insights gained could inform the rational design of therapeutic chitinase variants optimized for specific pathological conditions. By integrating mechanistic framework of Díaz *et al.* (37) and structural data of Olland *et al.* (38), our study provides a comprehensive perspective on the interplay between structural evolution and functional adaptation in Chia enzymes. This understanding enhances our knowledge of chitinase functionality and opens avenues for engineering Chia variants tailored for therapeutic applications.

Recent research elucidating a type 2 immune circuit in the stomach, which controls mammalian adaptation to dietary chitin (40), underscores the relevance of our study in the context of evolutionary biology and human health. This immune circuit highlights the intricate relationship between mammals and dietary chitin, suggesting that mammals, including humans, have evolved specialized mechanisms to tolerate and respond to chitin-rich diets. Integrating these findings with our research on chitinase hyperactivation provides a comprehensive understanding of the evolutionary dynamics underlying mammalian adaptation to dietary and environmental challenges.

Insights from this research may inspire the development of innovative treatments for diseases linked to chitin deposition, such as parasitic infections (7, 41), inflammatory bowel diseases (42), and other conditions associated with chitin accumulation. The enhanced chitinase activity could lead to more effective degradation of pathological chitin deposits, offering a novel therapeutic strategy.

By leveraging evolutionary principles, our research contributes to precision medicine, aiming to tailor treatments based on individual genetic and environmental factors. The insights gained from the evolutionary dynamics of chitinase genes and their interaction with immune pathways deepens our understanding of chitin metabolism and immune responses. This knowledge enhances our comprehension of host–pathogen interactions and holds promise for developing targeted therapies for chitin-related disorders.

In summary, our findings highlight the potential of protein engineering to enhance the functionality of human enzymes, providing a foundation for translational applications in medicine. By integrating biochemical, structural, and evolutionary insights, we pave the way for innovative treatments that address the underlying mechanisms of chitin-associated diseases, ultimately improving patient outcomes and public health.

## Experimental procedures

### Construction of human and monkey Chia expression vectors for *Escherichia coli* expression

Plasmid DNAs encoding human and monkey Chia were amplified by PCR using KOD Plus DNA polymerase (Toyobo) and specific oligonucleotide primers (Eurofins Genomics) that were designed with BamHI and XhoI restriction sites as anchors (Table S2) (19, 20, 25). The amplified human and monkey Chia cDNAs were digested with BamHI and XhoI and subsequently cloned into the corresponding sites of pET22b/pre-Protein A-dog Chia-V5-His (20). The complete plasmid DNA sequence was confirmed through sequencing (Eurofins Genomics).

### Construction of chimeric proteins and preparation of human Chia mutant proteins

Given the similar exon structures of both molecules at the nucleotide level, chimeric proteins were constructed by fusing two units at the exon junctions 3 to 5, 6 to 7, 8 to 10, and 11. This fusion was performed as described (20, 25, 43) (Tables S1 and S2). Human Chia mutant proteins were prepared using PCR with the appropriate primers as previously described (25).

### Preparation of recombinant human and monkey Chia proteins expressed in *E. coli*

Recombinant Protein A-human Chia-V5-His and Protein A-monkey Chia-V5-His expressed in *E. coli* were prepared and purified using IgG Sepharose (GE Healthcare), following established procedures (20).

### SDS-PAGE and Western blot

Protein fractions were subjected to standard SDS-PAGE and subsequent Western blot using an anti-V5-HRP mAb (Thermo Fisher Scientific). Immunoblots were analyzed and quantified using the Luminescent Image Analyzer (ImageQuant LAS 4000, GE Healthcare) following the manufacturer's instructions.

### Chitinase enzymatic assays

Chitinase enzyme activity was determined using the fluorogenic substrate 4-MU-(GlcNAc)_2_ hydrate (Sigma-Aldrich) as a substrate in McIlvaine’s buffer (0.1 M citric acid and 0.2 M Na_2_HPO_4_; pH 2.0, 5.0, 7.0) at 37 °C for 30 min, as previously described (20). The fluorescence of released 4-MU was measured using the GloMax Discover System GM3000 (Promega) with excitation at 365 nm and emission at 415 to 445 nm.

The initial rate of 4-MU-(GlcNAc)_2_ hydrolysis was measured in triplicate at 37 °C. Kinetic parameters, *k*_cat_ and *K*_m_ for 4-MU-(GlcNAc)_2_, were calculated assuming a Michaelis–Menten kinetic model as described previously (34). The initial rate of colloidal chitin hydrolysis was measured in triplicate at 37 °C.

### Analysis of chitooligosaccharides by FACE

The substrate was colloidal chitin (P-CHITN, Megazyme). Chitin substrates (1 mg/reaction) were incubated with recombinant Chia proteins at pH 2.0, 5.0, or 7.0 in McIlvaine's buffer (0.1 M citric acid and 0.2 M Na_2_HPO4) at 37 °C for 1 h. The degradation products were labeled and separated using FACE, following established protocols (36).

Kinetic parameters, Vmax and Km for colloidal chitin, were calculated assuming a Michaelis–Menten kinetic model as described previously at pH 5.0 at 37 °C for 30 min (44).

## Data availability

Data supporting the reported results will be available from the corresponding author (F. O.).

## Supporting information

This article contains supporting information.

## Conflict of interest

K. O., P. O. B., and F. O. are inventors of a patent application EP23212155.8 for the mutants described herein and their use in treating human diseases. The other authors declare that they have no conflicts of interest with the contents of this article.
