# Supplementary material for: Hyperactivation of human acidic chitinase (Chia) for potential medical use
Source: J Biol Chem. 2024 Dec 18;301(1):108100. doi: 10.1016/j.jbc.2024.108100 (PMC11773036; doi:10.1016/j.jbc.2024.108100)
Supplement: Supporting information [file mmc1.pdf]

Supporting information

Hyperactivation of human acidic chitinase (Chia) for potential medical use

Kazuaki Okawa, Masashi Kijima, Mana Ishii, Nanako Maeda, Yudai Yasumura, Masayoshi Sakaguchi, Masahiro Kimura, Maiko Uehara, Eri Tabata, Peter O. Bauer, and Fumitaka Oyama

Supplementary Figs. S1-S8 and Supplementary Tables S1 and S2.

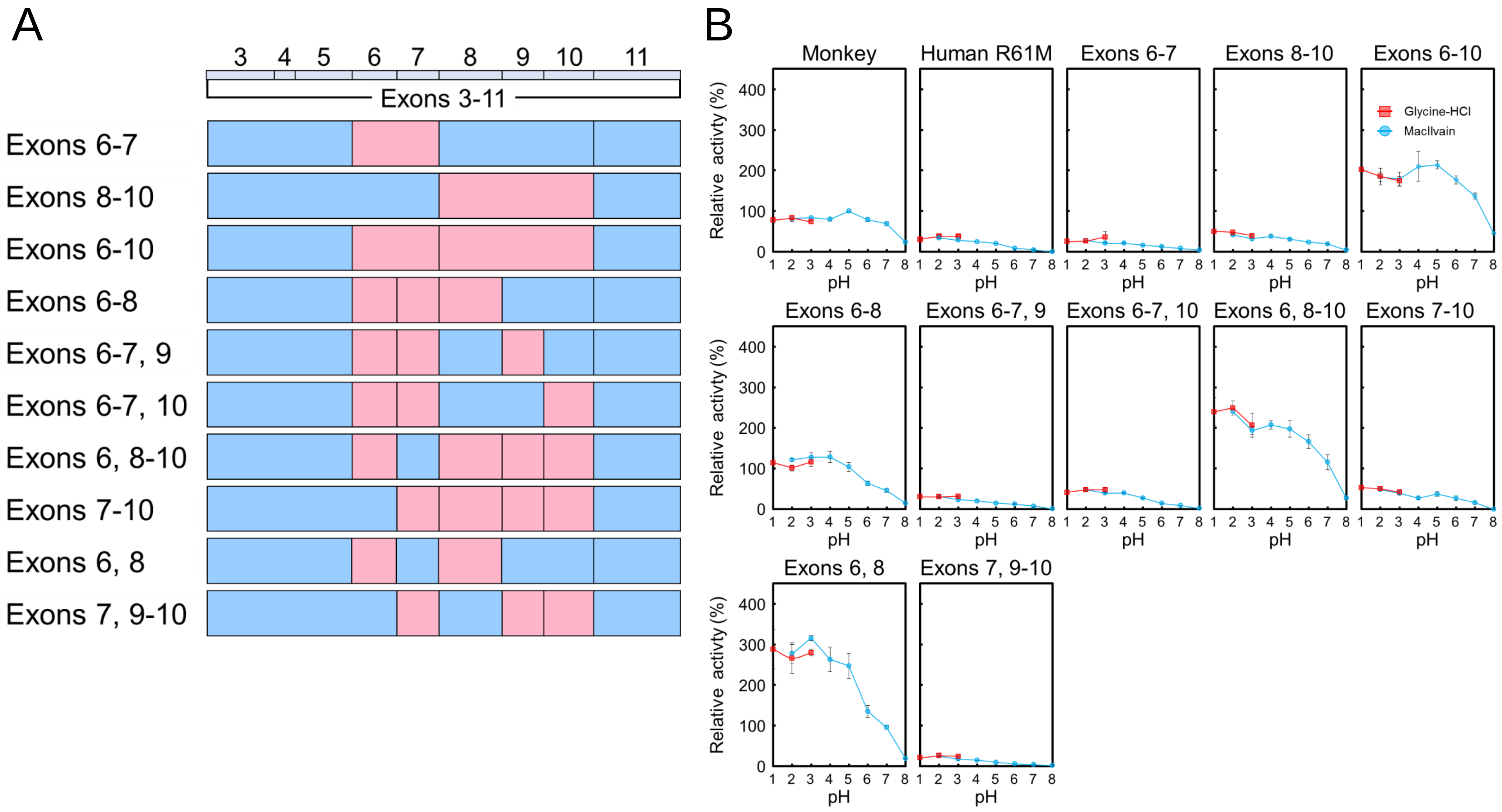

Supplementary Fig. S1. Investigation of the regions of monkey CHIA involved in the hyperactivation of human CHIA using chimeras prepared with monkey CHIA substituting multiple human CHIA exons. (A) Generated chimeras introducing multiple monkey Chia exons into human Chia R61M to determine the exons responsible for human Chia hyperactivation. (B) Chitinolytic activities of monkey Chia, human Chia R61M, and Chia chimeric proteins. Only the chimeras in which exons 6 and 8 of human Chia R61M were replaced with monkey CHIA showed hyperactivation.

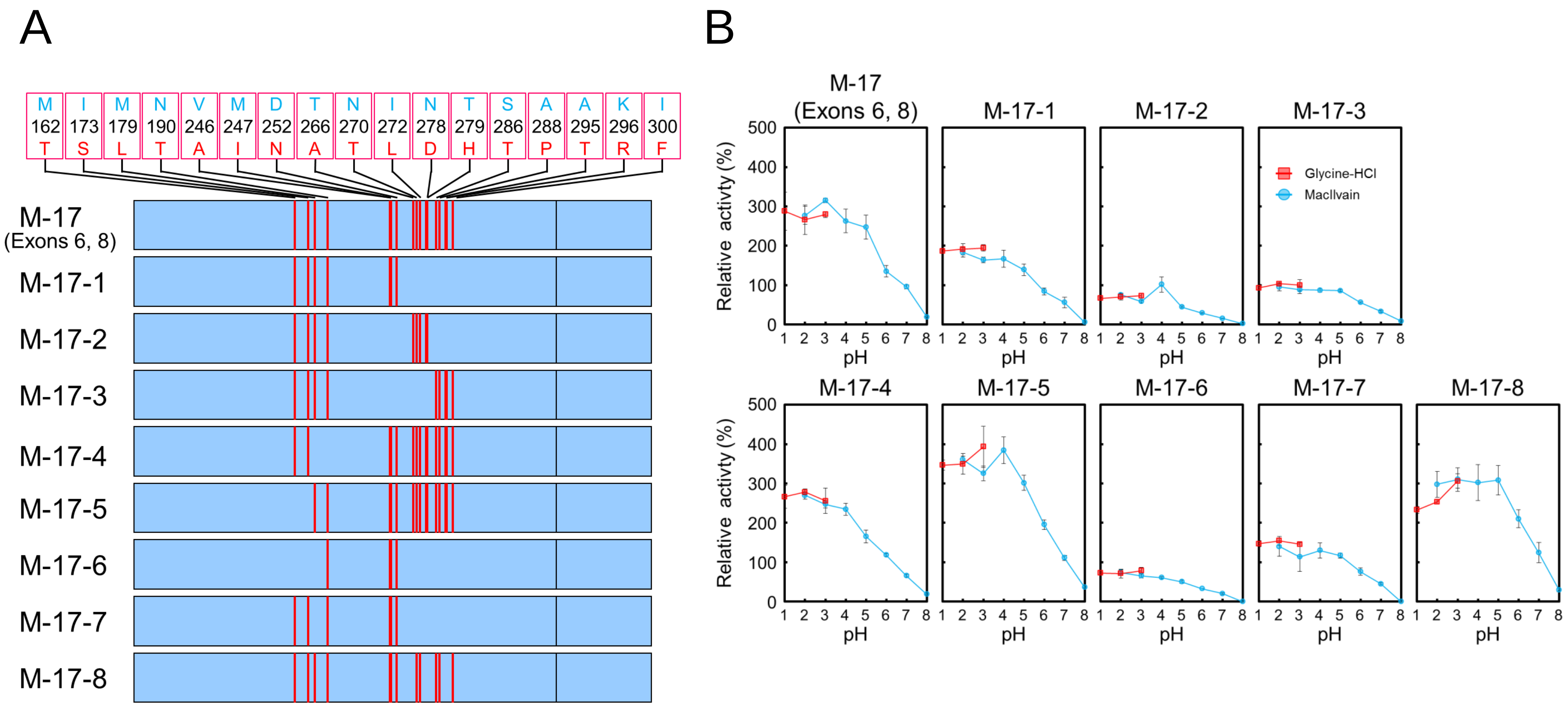

Supplementary Fig. S2. Identification of the amino acids involved in the hyperactivation of human Chia. (A) Mutants (M-17-1 to M-17-8) were generated with various combinations of monkey Chia-derived amino acids present in M-17. (B) Chitinolytic activities of mutants. Mutants M-17-5 and M-17-8 exhibited particularly high chitinase activity.

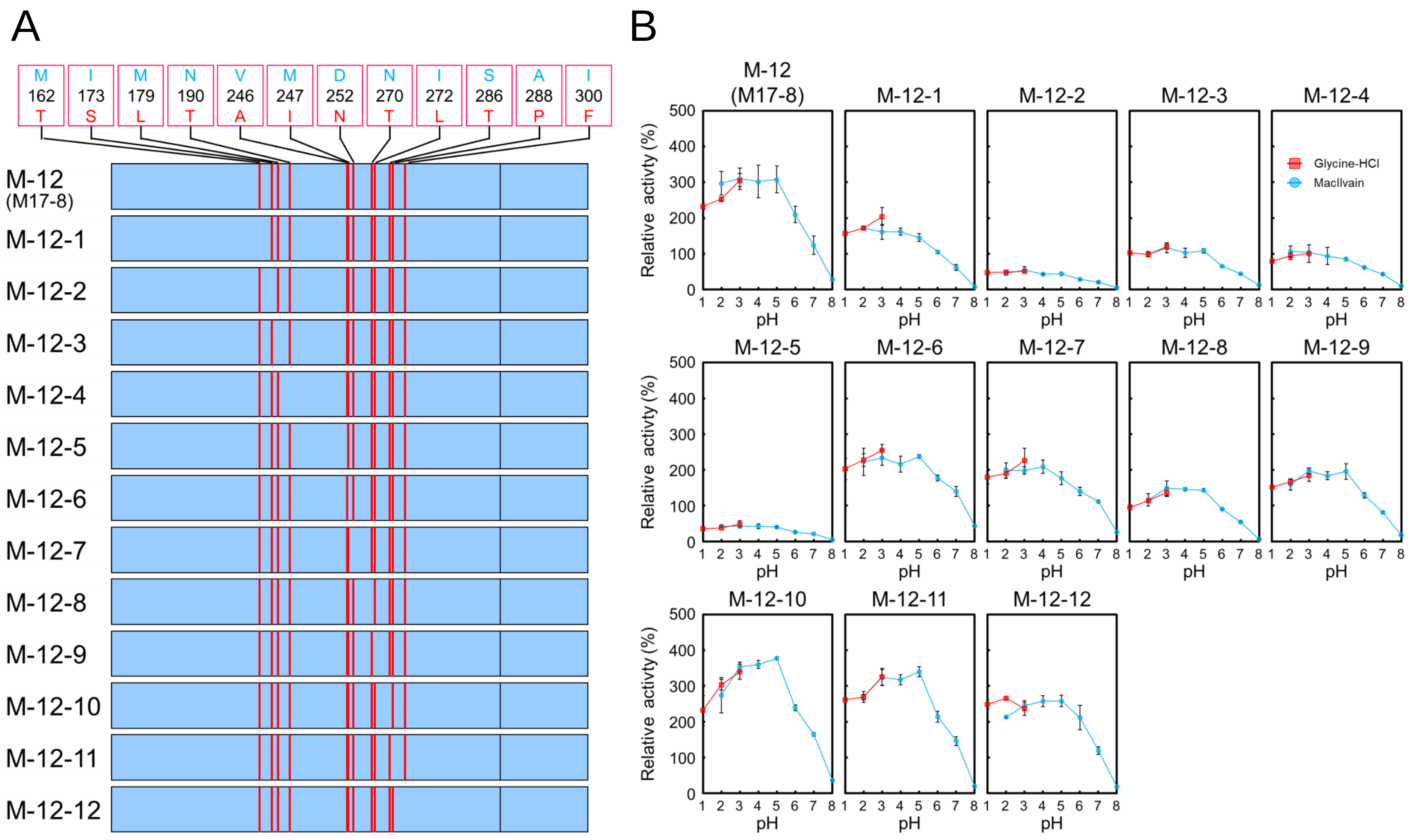

Supplementary Fig. S3. Further Identification of the amino acids involved in the hyperactivation of human Chia. (A) Amino acid mutants (M-12-1 to M-12-12) were generated by substituting each amino acid M-12 with the corresponding residue from human CHIA, one at a time. (B) Chitinolytic activities of the mutants. Mutants M-12-1 to M-12-12 exhibited higher chitin-degrading activity than monkey CHIA, except for M-12-2 and M-12-5.

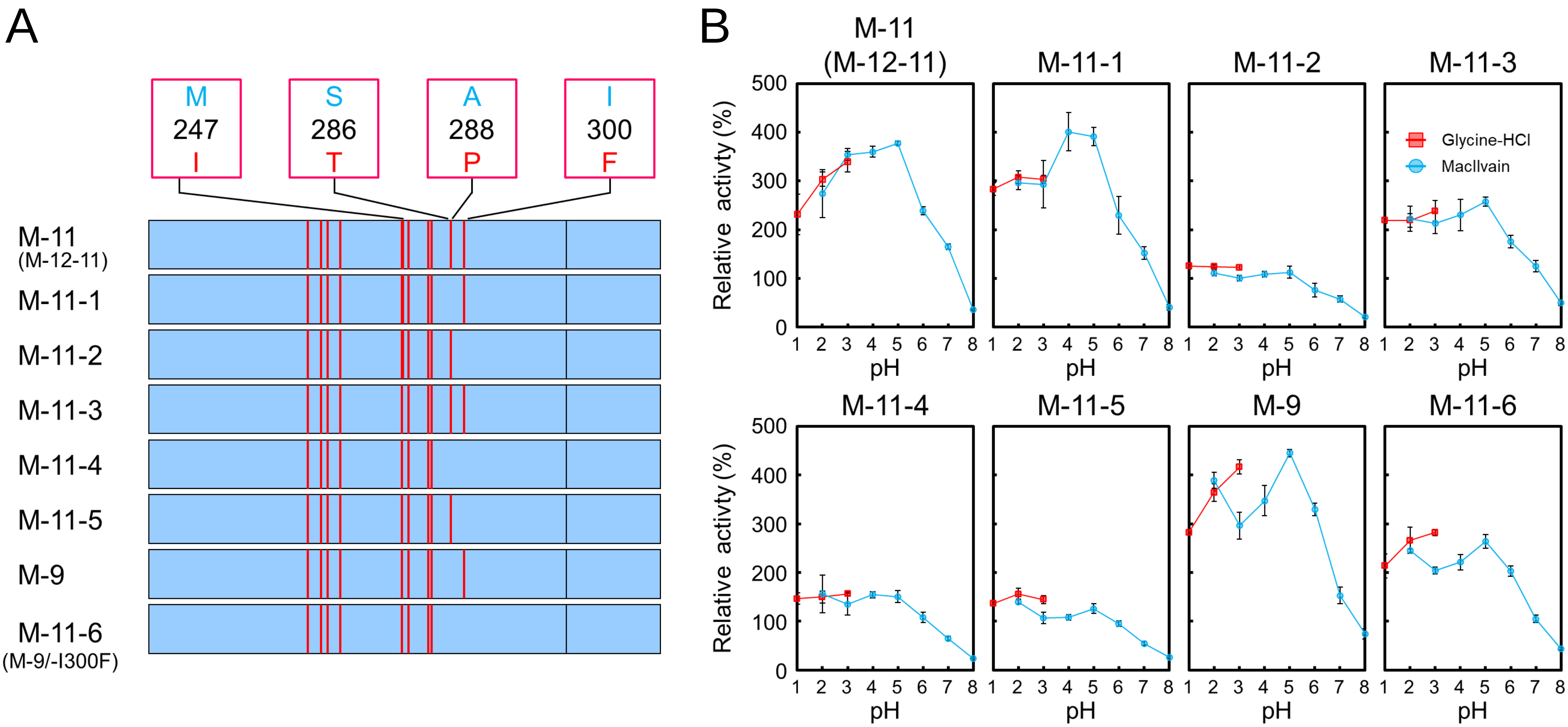

Supplementary Fig. S4. Further Identification of the amino acids involved in the hyperactivation of human Chia. (A) The amino acid substitutions in M-11 were reverted to human Chia-derived amino acids in various combinations to assess whether hyperactivation is maintained. (B) Chitinolytic activities of the mutants. All prepared human Chia mutants (M-11-1 to M-11-6 and M-9) exhibited higher activity than monkey Chia. Particularly, M-9 showed maximal hyperactivation.

A

	Exon 6				Exon 8				
	162	173	179	190	246	252	270	272	300
M-9	T	S	L	T	A	N	T	L	F
5 Mutation	M	I	M	N	A	N	T	L	F
5 Mutation/+M162T	T	I	M	N	A	N	T	L	F
5 Mutation/+I173S (M-6)	M	S	M	N	A	N	T	L	F
5 Mutation/+M179L	M	I	L	N	A	N	T	L	F
5 Mutation/+N190T	M	I	M	T	A	N	T	L	F
4 Mutation	T	S	L	T	V	D	N	I	I
4 Mutation/+V246A	T	S	L	T	A	D	N	I	I
4 Mutation/+D252N	T	S	L	T	V	N	N	I	I
4 Mutation/+N270T	T	S	L	T	V	D	T	I	I
4 Mutation/+I272L	T	S	L	T	V	D	N	L	I
4 Mutation/+I300F	T	S	L	T	V	D	N	I	F

B

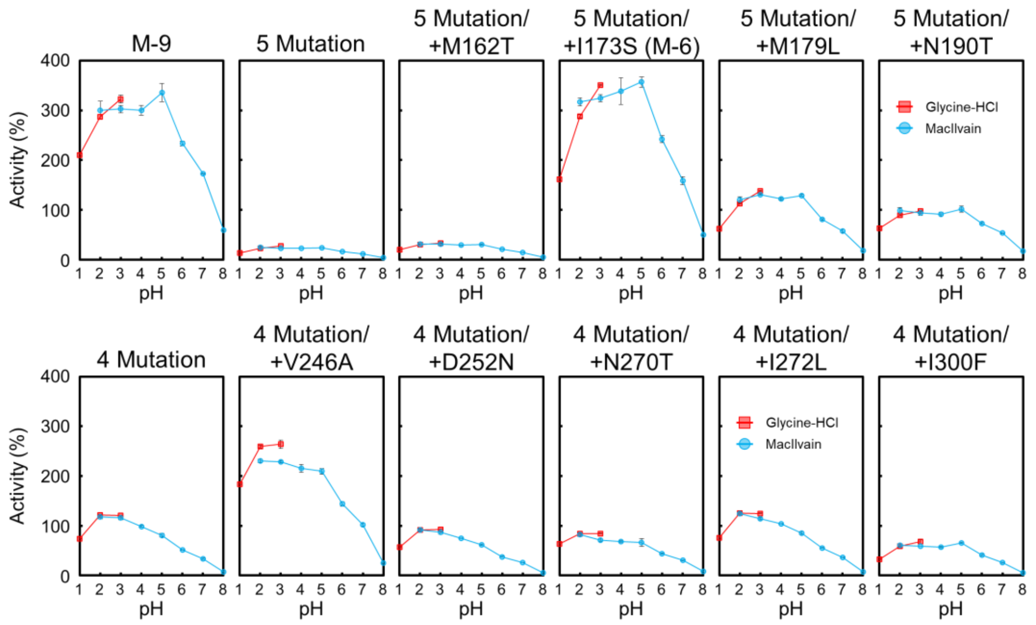

Supplementary Fig. S5. I173S and V246A are required for human Chia hyperactivation. (A) Amino acid substitutions in M-9 were reverted to human Chia-derived amino acids in various combinations to assess whether hyperactivation is maintained. (B) The generated human Chia mutant exhibited higher activity than monkey Chia when I173S and V246A were substituted simultaneously.

A

	Exon 6 162	173	179	190	Exon 8 246	252	270	272	300
M-9	T	S	L	T	A	N	T	L	F
M-2	M	S	M	N	A	D	N	I	I
M-2/+N270T	M	S	M	N	A	D	T	I	I
M-2/+N270T, I300F	M	S	M	N	A	D	T	I	F
M-6	M	S	M	N	A	N	T	L	F

	Exon 6 162	173	179	190	Exon 8 246	252	270	272	300
M-9	T	S	L	T	A	N	T	L	F
M-2	M	S	M	N	A	D	N	I	I
M-2/+M179L	M	S	L	N	A	D	N	I	I
M-2/+N190T	M	S	M	T	A	D	N	I	I
M-4	M	S	L	T	A	D	N	I	I

B

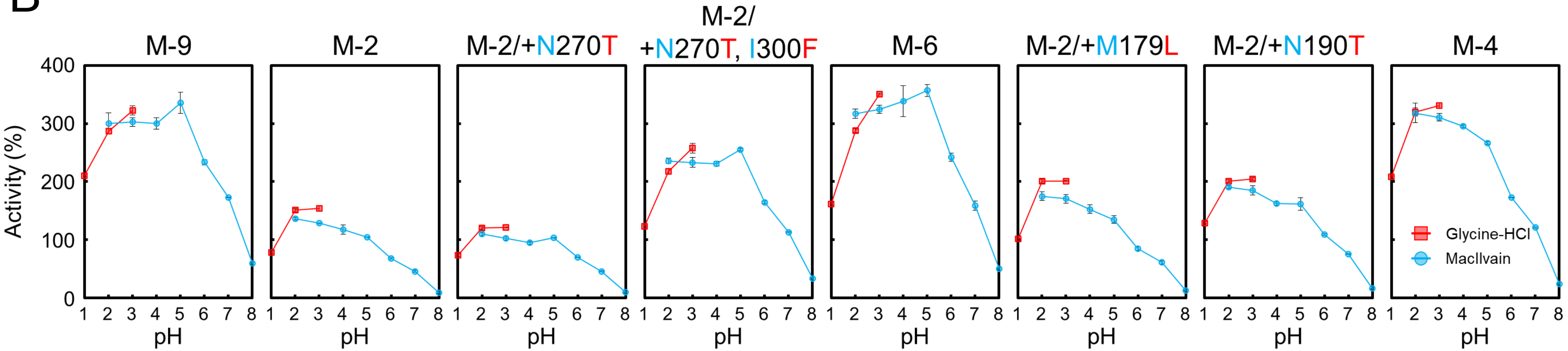

Supplementary Fig. S6. Improvement of function by introducing amino acid substitutions into M-2. (A) Amino acids derived from monkey Chia were introduced into M-2 to assess hyperactivation. (B) The introduction of amino acid substitutions into M-2 gradually increased chitinase activity.

Scientific name	Exon 6				Exon 8					Family
	162 ···	173 ···	179 ···	190	246 ···	252 ···	270 ···	272 ···	300	
Homo_sapiens	M	I	M	N	V	D	N	I	I	
Pan_troglodytes	M	I	M	N	V	D	K	I	I	
Pan_paniscus	M	I	M	N	V	D	N	I	I	*Hominidae*
Gorilla_gorilla_gorilla	M	I	M	N	V	D	N	I	I	
Pongo_abelii	M	I	M	N	V	D	T	I	T	
Nomascus_leucogenys	M	I	M	N	V	D	T	I	I	*Hylobatidae*
Macaca_fascicularis	T	S	L	T	A	N	T	L	F	
Allochrocebus_lhoesti	T	S	L	T	A	D	T	L	F	*Cercopithecidae*
Rhinopithecus_roxellana	T	S	L	T	A	D	T	L	F	
Plecturocebus_moloch	M	I	M	N	A	D	T	L	F	*Pitheciidae*
Cebus_imitator	M	I	M	N	V	D	T	T	F	*Cebidae*
Sapajus_apella	M	I	M	N	A	D	T	L	F	
Callithrix_jacchus	M	I	M	N	V	D	T	L	F	*Callitrichidae*
Carlito_syrichta_Chia2	M	I	M	N	A	D	T	I	F	*Tarsiidae*
Carlito_syrichta_Chia3	M	I	M	N	A	D	T	I	F	
Mus_musculus	M	S	M	N	V	N	T	I	F	*Muridae*
Tupaia	M	I	M	N	A	D	T	L	F	*Tupaiidae*
Myotis_lucifugus	M	V	M	N	I	D	T	I	F	
Eptesicus_fuscus	M	V	M	N	I	D	T	I	F	*Vespertilionidae*
Myotis_brandtii	M	V	M	N	I	D	T	I	F	
Sus_scrofa	M	T	L	N	A	D	T	L	F	*Suidae*
Odocoileus_virginianus_texanus	T	T	L	N	A	N	S	T	F	*Cervidae*
Camelus_dromedarius	M	T	L	N	A	D	T	I	F	*Camelidae*
Capra_hircus	T	T	L	N	A	S	N	I	F	*Bovidae*
Elephantulus_edwardii_Chia1	M	T	L	T	A	N	T	T	T	*Soricidae*
Elephantulus_edwardii_Chia2	M	V	M	N	A	D	T	I	F	
Suricata_suricatta	M	I	M	N	V	E	T	I	F	*Carnivora*
Gallus_gallus	M	V	M	N	A	S	S	I	F	*Phasianidae*

Supplementary Fig. S7. Comparison of amino acids in Chia exons 6 and 8 involved overactivation in various species.

```
Homo_sapiens         22   YQLTCYFTNWAQYRPGLGRFMPDNIDPCLCTHLIYAFAGRQNNEITTIEWNDVTLYQAFN 81
M-9                  22   YQLTCYFTNWAQYRPGLGRFMPDDINPCLCTHLIYAFAGMQNNEITTIEWNDVTLYQAFN 81
Macaca_fascicularis  22   YQLTCYFSNWAQYRPGLGRFMPDDIDPCLCTHLIYAFAGMQNNKITTIEWNDVTLYQAFN 81
Mus_musculus         22   YNLICYFTNWAQYRPGLGSFKPDDINPCLCTHLIYAFAGMQNNEITTIEWNDVTLYKAFN 81

Homo_sapiens         82   GLKNKNSQLKTLLAIGGWNFGTAPFTAMVSTPENRQTFITSVIKFLRQYEFDGLDFDWEY 141
M-9                  82   GLKNKNSQLKTLLAIGGWNFGTAPFTAMVSTPENRQTFITSVIKFLRQYEFDGLDFDWEY 141
Macaca_fascicularis  82   GLKNKNSQLKTLLAIGGWNFGTAPFTAMVSTPANRQTFINSVIEFLRQYEFDGLDFDWEY 141
Mus_musculus         82   DLKNRNSKLKTLLAIGGWNFGTAPFTTMVSTSQNRQTFITSVIKFLRQYGFDGLDLDWEY 141

                                             162        173   179        190
Homo_sapiens         142  PGSRGSPPQDKHLFTVLVQEMREAFEQEAKQINKPRLMVTAAVAAGISNIQSGYEIPQLS 201
M-9                  142  PGSRGSPPQDKHLFTVLVQETREAFEQEAKQSNKPRLLVTAAVAAGISTIQSGYEIPQLS 201
Macaca_fascicularis  142  PGSRGSPSQDKHLFTVLVQETREAFEQEAKQSNKPRLLVTAAVAAGISTIQSGYEIPQLS 201
Mus_musculus         142  PGSRGSPPQDKHLFTVLVKEMREAFEQEAIESNRPRLMVTAAVAGGISNIQAGYEIPELS 201

                                                                     246   252
Homo_sapiens         202  QYLDYIHVMTYDLHGSWEGYTGENSPLYKYPTDTGSNAYLNVDYVMNYWKDNGAPAEKLI 261
M-9                  202  QYLDYIHVMTYDLHGSWEGYTGENSPLYKYPTDTGSNAYLNVDYAMNYWKNNGAPAEKLI 261
Macaca_fascicularis  202  QYLDYIHVMTYDLHGPWEGYTGENSPLYKYPTDTGSNAYLNVDYAINYWKNNGAPAEKLI 261
Mus_musculus         202  KYLDFIHVMTYDLHGSWEGYTGENSPLYKYPTETGSNAYLNVDYVMNYWKNNGAPAEKLI 261

                                 270 272                       300
Homo_sapiens         262  VGFPTYGHNFILSNPSNTGIGAPTSGAGPAGPYAKESGIWAYYEICTFLKNGATQGWDAP 321
M-9                  262  VGFPTYGHTFLLSNPSNTGIGAPTSGAGPAGPYAKESGFWAYYEICTFLKNGATQGWDAP 321
Macaca_fascicularis  262  VGFPAYGHTFLLSNPSDHGIGAPTTGPGPAGPYTRESGFWAYYEICTFLKNGATEVWEAN 321
Mus_musculus         262  VGFPEYGHTFILRNPSDNGIGAPTSGDGPAGAYTRQAGFWAYYEICTFLRSGATEVWDAS 321

Homo_sapiens         322  QEVPYAYQGNVWVGYDNIKSFDIKAQWLKHNKFGGAMVWAIDLDDFTGTFCNQGKFPLIS 381
M-9                  322  QEVPYAYQGNVWVGYDNIKSFDIKAQWLKHNKFGGAMVWAIDLDDFTGTFCNQGKFPLIS 381
Macaca_fascicularis  322  EDVPYAYKGNEWLGYDNTKSFQIKADWLKKNNFGGAMVWAIDLDDFTGTFCNEGKFPLIT 381
Mus_musculus         322  QEVPYAYKANEWLGYDNIKSFSVKAQWLKQNNFGGAMIWAIDLDDFTGSFCDQGKFPLTS 381

Homo_sapiens         382  TLKKALGLQSASCTAPAQPIEPITAAPSGSGNGSGSSSSGGSSGGSGFCAVRANGLYPVA 441
M-9                  382  TLKKALGLQSASCTAPAQPIEPITAAPSGSGNGSGSSSSGGSSGGSGFCAVRANGLYPVA 441
Macaca_fascicularis  382  TLKDALGLQSTSCKAPAQPITPITEAPVTG-SVSHSGSSGGSPSDSEFCANKANGLYPDP 440
Mus_musculus         382  TLNKALGISTEGCTAPDVPSEPVTTPPG---SGSGGGSSGGSSGGSGFCADKADGLYPVA 438

Homo_sapiens         442  NNRNAFWHCVNGVTYQQNCQAGLVFDTSCDCCNWA 476
M-9                  442  NNRNAFWHCVNGVTYQQNCQAGLVFDTSCDCCNWA 476
Macaca_fascicularis  441  TDKNAFYNCANGKTFIQHCQAGLVFEASCSCCSW- 474
Mus_musculus         439  DDRNAFWQCINGITYQQHCQAGLVFDTSCNCCNWP 473
```

Supplementary Fig. S8. Comparison of amino acid sequences of human, monkey, and mouse Chia.

Supplementary Table S1. Combination of primer and template to prepare each chimera by PCR.

Products name	Part	Template	Forward	Reverse
Monkey Chia		Monkey Chia	Macaca_Bam_Fw	Macaca_ XhoI_Rv
Human Chia		Human Chia	Hum_Bam_Fw	Hum_XhoI_Rv
C1	1	Monkey Chia	pEZZ18_Fw2533	C1_Rv
	2	Human Chia R61M	C1_Fw	Sal_BGH_Rv
	Final	C1 Part 1 and 2	pEZZ18_Fw2533	Sal_BGH_Rv
C2	1	Monkey Chia	pEZZ18_Fw2533	C2/C17/C18_Rv
	2	Human Chia R61M	C2/C17/C18_Fw	Sal_BGH_Rv
	Final	C2 Part 1 and 2	pEZZ18_Fw2533	Sal_BGH_Rv
C2	1	Monkey Chia	pEZZ18_Fw2533	C3_Rv
	2	Human Chia R61M	C3_Fw	Sal_BGH_Rv
	Final	C3 Part 1 and 2	pEZZ18_Fw2533	Sal_BGH_Rv
C4	1	Human Chia R61M	pEZZ18_Fw2533	C4_Rv
	2	Monkey Chia	C4_Fw	Sal_BGH_Rv
	Final	C4 Part 1 and 2	pEZZ18_Fw2533	Sal_BGH_Rv
C5	1	Human Chia R61M	pEZZ18_Fw2533	C5/C14/C19_Rv
	2	Monkey Chia	C5/C14/C19_Fw	Sal_BGH_Rv
	Final	C5 Part 1 and 2	pEZZ18_Fw2533	Sal_BGH_Rv
C6	1	Human Chia R61M	pEZZ18_Fw2533	C6/C13/C15_Rv
	2	Monkey Chia	C6/C13/C15_Fw	Sal_BGH_Rv
	Final	C6 Part 1 and 2	pEZZ18_Fw2533	Sal_BGH_Rv
C7	1	C6	pEZZ18_Fw2533	C7/C12_Rv
	2	Human Chia R61M	C7/C12_Fw	Sal_BGH_Rv
	Final	C7 Part 1 and 2	pEZZ18_Fw2533	Sal_BGH_Rv
C8	1	Human Chia R61M	pEZZ18_Fw2533	C8/C20_Rv
	2	C2	C8/C20_Fw	Sal_BGH_Rv
	Final	C8 Part 1 and 2	pEZZ18_Fw2533	Sal_BGH_Rv
C9	1	C5	pEZZ18_Fw2533	C9/C16_Rv
	2	Human Chia R61M	C9/C16_Fw	Sal_BGH_Rv
	Final	C9 Part 1 and 2	pEZZ18_Fw2533	Sal_BGH_Rv
C10	1	Human Chia R61M	pEZZ18_Fw2533	C10/C21_Rv1
	2	Monkey Chia	C10/C21_Fw1	C10_Rv2
	3	Human Chia R61M	C10_Fw2	Sal_BGH_Rv

	Final	C10 Part 1, 2, and 3	pEZZ18_Fw2533	Sal_BGH_Rv
C11	1	Human Chia R61M	pEZZ18_Fw2533	C11_Rv
	2	C1	C11_Fw	Sal_BGH_Rv
	Final	C11 Part 1 and 2	pEZZ18_Fw2533	Sal_BGH_Rv
C12	1	C7	pEZZ18_Fw2533	C7/C12_Rv
	2	C9	C7/C12_Fw	Sal_BGH_Rv
	Final	C12 Part 1 and 2	pEZZ18_Fw2533	Sal_BGH_Rv
C13	1	Human Chia R61M	pEZZ18_Fw2533	C6/C13/C15_Rv
	2	C2	C6/C13/C15_Fw	Sal_BGH_Rv
	Final	C13 Part 1 and 2	pEZZ18_Fw2533	Sal_BGH_Rv
C14	1	Human Chia R61M	pEZZ18_Fw2533	C5/C14/C19_Rv
	2	C1	C5/C14/C19_Fw	Sal_BGH_Rv
	Final	C14 Part 1 and 2	pEZZ18_Fw2533	Sal_BGH_Rv
C15	1	Human Chia R61M	pEZZ18_Fw2533	C6/C13/C15_Rv
	2	C1	C6/C13/C15_Fw	Sal_BGH_Rv
	Final	C14 Part 1 and 2	pEZZ18_Fw2533	Sal_BGH_Rv
C16	1	C6	pEZZ18_Fw2533	C9/C16_Rv
	2	Human Chia R61M	C9/C16_Fw	Sal_BGH_Rv
	Final	C15 Part 1 and 2	pEZZ18_Fw2533	Sal_BGH_Rv
C17	1	C13	pEZZ18_Fw2533	C2/C17/C18_Rv
	2	C10	C2/C17/C18_Fw	Sal_BGH_Rv
	Final	C17 Part 1 and 2	pEZZ18_Fw2533	Sal_BGH_Rv
C18	1	C13	pEZZ18_Fw2533	C2/C17/C18_Rv
	2	C11	C2/C17/C18_Fw	Sal_BGH_Rv
	Final	C17 Part 1 and 2	pEZZ18_Fw2533	Sal_BGH_Rv
C19	1	C7	pEZZ18_Fw2533	C5/C14/C19_Rv
	2	C13	C5/C14/C19_Fw	Sal_BGH_Rv
	Final	C17 Part 1 and 2	pEZZ18_Fw2533	Sal_BGH_Rv
C20	1	Human Chia R61M	pEZZ18_Fw2533	C8/C20_Rv
	2	C14	C8/C20_Fw	Sal_BGH_Rv
	Final	C20 Part 1 and 2	pEZZ18_Fw2533	Sal_BGH_Rv
C21	1	C8	pEZZ18_Fw2533	C10/C21_Rv1
	2	C14	C10/C21_Fw1	Sal_BGH_Rv
	Final	C21 Part 1 and 2	pEZZ18_Fw2533	Sal_BGH_Rv

M-17-1	1	Human Chia R61M	pEZZ18_Fw2533	M-17-1/2/3/8_Ex6_Rv
	2	Human Chia R61M	M-17-1/2/3/8_Ex6_Fw	M-17-1/4/5/6/7/8_Ex8_Rv
	3	Human Chia R61M	M-17-1/4/5/6/7/8_Ex8_Fw	Sal_BGH_Rv
	Final	M-17-1 Part 1, 2, and 3	pEZZ18_Fw2533	Sal_BGH_Rv
M-17-2	1	Human Chia R61M	pEZZ18_Fw2533	M-17-1/2/3/8_Ex6_Rv
	2	Human Chia R61M	M-17-1/2/3/8_Ex6_Fw	M-17-2_Ex8_Rv
	3	Human Chia R61M	M-17-2/4/5_Ex8_Fw	Sal_BGH_Rv
	Final	M-17-2 Part 1, 2, and 3	pEZZ18_Fw2533	Sal_BGH_Rv
M-17-3	1	Human Chia R61M	pEZZ18_Fw2533	M-17-1/2/3/8_Ex6_Rv
	2	Human Chia R61M	M-17-1/2/3/8_Ex6_Fw	M-17-3_Ex8_Rv
	3	Human Chia R61M	M-17-3/4/5_Ex8_Fw	Sal_BGH_Rv
	Final	M-17-3 Part 1, 2, and 3	pEZZ18_Fw2533	Sal_BGH_Rv
M-17-4	1	Human Chia R61M	pEZZ18_Fw2533	M-17-4_Ex6_Rv
	2	Human Chia R61M	M-17-4_Ex6_Fw	M-17-1/4/5/6/7/8_Ex8_Rv
	3	Human Chia R61M	M-17-3/4/5_Ex8_Fw	Sal_BGH_Rv
	4	M-17-4 Part 3	M-17-2/4/5_Ex8_Fw	Sal_BGH_Rv
	5	M-17-4 Part 4	M-17-1/4/5/6/7/8_Ex8_Fw	Sal_BGH_Rv
	Final	M-17-4 Part 1, 2, and 5	pEZZ18_Fw2533	Sal_BGH_Rv
M-17-5	1	Human Chia R61M	pEZZ18_Fw2533	M-17-5_Ex6_Rv
	2	Human Chia R61M	M-17-5/6_Ex6_Fw	M-17-1/4/5/6/7/8_Ex8_Rv
	3	Human Chia R61M	M-17-3/4/5_Ex8_Fw	Sal_BGH_Rv
	4	M-17-5 Part 3	M-17-2/4/5_Ex8_Fw	Sal_BGH_Rv
	5	M-17-5 Part 4	M-17-1/4/5/6/7/8_Ex8_Fw	Sal_BGH_Rv
	Final	M-17-5 Part 1, 2, and 5	pEZZ18_Fw2533	Sal_BGH_Rv
M-17-6	1	Human Chia R61M	pEZZ18_Fw2533	M-17-6_Ex6_Rv
	2	Human Chia R61M	M-17-5/6_Ex6_Fw	M-17-1/4/5/6/7/8_Ex8_Rv
	3	Human Chia R61M	M-17-1/4/5/6/7/8_Ex8_Fw	Sal_BGH_Rv
	Final	M-17-6 Part 1, 2, and 3	pEZZ18_Fw2533	Sal_BGH_Rv
M-17-7	1	Human Chia R61M	pEZZ18_Fw2533	M-17-7_Ex6_Rv
	2	Human Chia R61M	M-17-7_Ex6_Fw	M-17-1/4/5/6/7/8_Ex8_Rv
	3	Human Chia R61M	M-17-1/4/5/6/7/8_Ex8_Fw	Sal_BGH_Rv
	Final	M-17-7 Part 1, 2, and 3	pEZZ18_Fw2533	Sal_BGH_Rv
	1	Human Chia R61M	pEZZ18_Fw2533	M-17-1/2/3/8_Ex6_Rv
	2	Human Chia R61M	M-17-1/2/3/8_Ex6_Fw	M-17-1/4/5/6/7/8_Ex8_Rv

M-17-8 (M-12)	3	Human Chia R61M	M-17-8_Ex8_Fw1	Sal_BGH_Rv
	4	M-17-8 Part 3	M-17-8_Ex8_Fw2	Sal_BGH_Rv
	5	M-17-8 Part 4	M-17-1/4/5/6/7/8_Ex8_Fw	Sal_BGH_Rv
	Final	M-17-8 Part 1, 2, and 5	pEZZ18_Fw2533	Sal_BGH_Rv
M-12-1	1	M-17-8	pEZZ18_Fw2533	M-12-1_Rv
	2	M-17-8	M-12-1_Fw	Sal_BGH_Rv
	Final	M-12-1 Part 1 and 2	pEZZ18_Fw2533	Sal_BGH_Rv
M-12-2	1	M-17-8	pEZZ18_Fw2533	M-12-2_Rv
	2	M-17-8	M-12-2_Fw	Sal_BGH_Rv
	Final	M-12-2 Part 1 and 2	pEZZ18_Fw2533	Sal_BGH_Rv
M-12-3	1	M-17-8	pEZZ18_Fw2533	M-12-3_Rv
	2	M-17-8	M-12-3_Fw	Sal_BGH_Rv
	Final	M-12-3 Part 1 and 2	pEZZ18_Fw2533	Sal_BGH_Rv
M-12-4	1	M-17-8	pEZZ18_Fw2533	M-12-4_Rv
	2	M-17-8	M-12-4_Fw	Sal_BGH_Rv
	Final	M-12-4 Part 1 and 2	pEZZ18_Fw2533	Sal_BGH_Rv
M-12-5	1	M-17-8	pEZZ18_Fw2533	M-12-5_Rv
	2	M-17-8	M-12-5_Fw	Sal_BGH_Rv
	Final	M-12-5 Part 1 and 2	pEZZ18_Fw2533	Sal_BGH_Rv
M-12-6	1	M-17-8	pEZZ18_Fw2533	M-12-6/M-11-3_Rv
	2	M-17-8	M-12-6_Fw	Sal_BGH_Rv
	Final	M-12-6 Part 1 and 2	pEZZ18_Fw2533	Sal_BGH_Rv
M-12-7	1	M-17-8	pEZZ18_Fw2533	M-12-7_Rv
	2	M-17-8	M-12-7_Fw	Sal_BGH_Rv
	Final	M-12-7 Part 1 and 2	pEZZ18_Fw2533	Sal_BGH_Rv
M-12-8	1	M-17-8	pEZZ18_Fw2533	M-12-8_Rv
	2	M-17-8	M-12-8_Fw	Sal_BGH_Rv
	Final	M-12-8 Part 1 and 2	pEZZ18_Fw2533	Sal_BGH_Rv
M-12-9	1	M-17-8	pEZZ18_Fw2533	M-12-9_Rv
	2	M-17-8	M-12-9_Fw	Sal_BGH_Rv
	Final	M-12-9 Part 1 and 2	pEZZ18_Fw2533	Sal_BGH_Rv
M-12-10	1	M-17-8	pEZZ18_Fw2533	M-12-10_Rv
	2	M-17-8	M-12-10_Fw	Sal_BGH_Rv
	Final	M-12-10 Part 1 and 2	pEZZ18_Fw2533	Sal_BGH_Rv

M-12-11<br>(M-11)	1	M-17-8	pEZZ18_Fw2533	M-12-11_Rv
	2	M-17-8	M-12-11_Fw	Sal_BGH_Rv
	Final	M-12-11 Part 1 and 2	pEZZ18_Fw2533	Sal_BGH_Rv
M-12-12	1	M-17-8	pEZZ18_Fw2533	M-12-12/M-11-2_Rv
	2	M-17-8	M-12-12/M-11-2_Fw	Sal_BGH_Rv
	Final	M-12-12 Part 1 and 2	pEZZ18_Fw2533	Sal_BGH_Rv
M-11-1	1	M-12-10	pEZZ18_Fw2533	M-11-1/6_Rv
	2	M-12-10	M-11-1/6_Fw	Sal_BGH_Rv
	Final	M-11-1 Part 1 and 2	pEZZ18_Fw2533	Sal_BGH_Rv
M-11-2	1	M-12-10	pEZZ18_Fw2533	M-12-12/M-11-2_Rv
	2	M-12-10	M-12-12/M-11-2_Fw	Sal_BGH_Rv
	Final	M-11-2 Part 1 and 2	pEZZ18_Fw2533	Sal_BGH_Rv
M-11-3	1	M-12-10	pEZZ18_Fw2533	M-12-6/M-11-3_Rv
	2	M-12-10	M16_Fw	Sal_BGH_Rv
	Final	M-11-3 Part 1 and 2	pEZZ18_Fw2533	Sal_BGH_Rv
M-11-4	1	M-12-10	pEZZ18_Fw2533	M-11-4/M-9_Rv
	2	M-12-10	M-11-4/M-9_Fw	Sal_BGH_Rv
	Final	M-11-4 Part 1 and 2	pEZZ18_Fw2533	Sal_BGH_Rv
M-11-5	1	M-12-6	pEZZ18_Fw2533	M-11-5_Rv
	2	M-12-6	M-11-5_Fw	Sal_BGH_Rv
	Final	M-11-5 Part 1 and 2	pEZZ18_Fw2533	Sal_BGH_Rv
M-9	1	M-12-6	pEZZ18_Fw2533	M-11-4/M-9_Rv
	2	M-12-6	M-11-4/M-9_Fw	Sal_BGH_Rv
	Final	M-9 Part 1 and 2	pEZZ18_Fw2533	Sal_BGH_Rv
M-11-6	1	M-12-6	pEZZ18_Fw2533	M-11-1/6_Rv
	2	M-12-6	M-11-1/6_Fw	Sal_BGH_Rv
	Final	M-11-6 Part 1 and 2	pEZZ18_Fw2533	Sal_BGH_Rv
M162T	1	Human Chia R61M	pEZZ18_Fw2533	M162T_Rv
	2	Human Chia R61M	M162T_Fw	Sal_BGH_Rv
	Final	M162T Part 1 and 2	pEZZ18_Fw2533	Sal_BGH_Rv
I173S	1	Human Chia R61M	pEZZ18_Fw2533	I173S_Rv
	2	Human Chia R61M	I173S_Fw	Sal_BGH_Rv
	Final	I173S Part 1 and 2	pEZZ18_Fw2533	Sal_BGH_Rv
	1	Human Chia R61M	pEZZ18_Fw2533	M179L_Rv

M179L	2	Human Chia R61M	M179L_Fw	Sal_BGH_Rv
	Final	M179L Part 1 and 2	pEZZ18_Fw2533	Sal_BGH_Rv
N190T	1	Human Chia R61M	pEZZ18_Fw2533	N190T_Rv
	2	Human Chia R61M	N190T_Fw	Sal_BGH_Rv
	Final	N190T Part 1 and 2	pEZZ18_Fw2533	Sal_BGH_Rv
V246A	1	Human Chia R61M	pEZZ18_Fw2533	V246A_Rv
	2	Human Chia R61M	V246A_Fw	Sal_BGH_Rv
	Final	V246A Part 1 and 2	pEZZ18_Fw2533	Sal_BGH_Rv
D252N	1	Human Chia R61M	pEZZ18_Fw2533	D252N_Rv
	2	Human Chia R61M	D252N_Fw	Sal_BGH_Rv
	Final	D252N Part 1 and 2	pEZZ18_Fw2533	Sal_BGH_Rv
N270T	1	Human Chia R61M	pEZZ18_Fw2533	N270T_Rv
	2	Human Chia R61M	N270T_Fw	Sal_BGH_Rv
	Final	N270T Part 1 and 2	pEZZ18_Fw2533	Sal_BGH_Rv
I272L	1	Human Chia R61M	pEZZ18_Fw2533	I272L_Rv
	2	Human Chia R61M	I272L_Fw	Sal_BGH_Rv
	Final	I272L Part 1 and 2	pEZZ18_Fw2533	Sal_BGH_Rv
I300F	1	Human Chia R61M	pEZZ18_Fw2533	I300F_Rv
	2	Human Chia R61M	I300F_Fw	Sal_BGH_Rv
	Final	I300F Part 1 and 2	pEZZ18_Fw2533	Sal_BGH_Rv
M-9/-T162M	1	M-9	pEZZ18_Fw2533	M-9/-T162M_Rv
	2	M-9	M-9/-T162M_Fw	Sal_BGH_Rv
	Final	M-9/-T162M Part 1 and 2	pEZZ18_Fw2533	Sal_BGH_Rv
M-9/-I173S	1	M-9	pEZZ18_Fw2533	M-9/-I173S_Rv
	2	M-9	M-9/-I173S_Fw	Sal_BGH_Rv
	Final	M-9/-I173S Part 1 and 2	pEZZ18_Fw2533	Sal_BGH_Rv
M-9/-L179M	1	M-9	pEZZ18_Fw2533	M-9/-L179M_Rv
	2	M-9	M-9/-L179M_Fw	Sal_BGH_Rv
	Final	M-9/-L179M Part 1 and 2	pEZZ18_Fw2533	Sal_BGH_Rv
M-9/-T190N	1	M-9	pEZZ18_Fw2533	M-9/-T190N_Rv
	2	M-9	M-9/-T190N_Fw	Sal_BGH_Rv
	Final	M-9/-T190N Part 1 and 2	pEZZ18_Fw2533	Sal_BGH_Rv
M-9/-A246V	1	M-9	pEZZ18_Fw2533	M-9/-A246V_Rv
	2	M-9	M-9/-A246V_Fw	Sal_BGH_Rv

	Final	M-9/-A246V Part 1 and 2	pEZZ18_Fw2533	Sal_BGH_Rv
M-9/-N252D	1	M-9	pEZZ18_Fw2533	M-9/-N252D_Rv
	2	M-9	M-9/-N252D_Fw	Sal_BGH_Rv
	Final	M-9/-N252D Part 1 and 2	pEZZ18_Fw2533	Sal_BGH_Rv
M-9/-T270N	1	M-9	pEZZ18_Fw2533	M-9/-T270N_Rv
	2	M-9	M-9/-T270N_Fw	Sal_BGH_Rv
	Final	M-9/-T270N Part 1 and 2	pEZZ18_Fw2533	Sal_BGH_Rv
M-9/-L272I	1	M-9	pEZZ18_Fw2533	M-9/-L272I_Rv
	2	M-9	M-9/-L272I_Fw	Sal_BGH_Rv
	Final	M-9/-L272I Part 1 and 2	pEZZ18_Fw2533	Sal_BGH_Rv
M-9/-F300I	1	M-9	pEZZ18_Fw2533	M-9/-F300I_Rv
	2	M-9	M-9/-F300I_Fw	Sal_BGH_Rv
	Final	M-9/-F300I Part 1 and 2	pEZZ18_Fw2533	Sal_BGH_Rv
M-2	1	I173S	pEZZ18_Fw2533	Human_Chia_Rv820
	2	V246A	Human_Chia_Fw735	Sal_BGH_Rv
	Final	M-2 Part 1 and 2	pEZZ18_Fw2533	Sal_BGH_Rv
M-4	1	M-9/-T162M	pEZZ18_Fw2533	Human_Chia_Rv820
	2	V246A	Human_Chia_Fw735	Sal_BGH_Rv
	Final	M-4 Part 1 and 2	pEZZ18_Fw2533	Sal_BGH_Rv
M-6	1	I173S	pEZZ18_Fw2533	Human_Chia_Rv820
	2	M-9	Human_Chia_Fw735	Sal_BGH_Rv
	Final	M-6 Part 1 and 2	pEZZ18_Fw2533	Sal_BGH_Rv
5 Mutation	1	Human Chia R61M	pEZZ18_Fw2533	Human_Chia_Rv820
	2	M-9	Human_Chia_Fw735	Sal_BGH_Rv
	Final	5 Mutation Part 1 and 2	pEZZ18_Fw2533	Sal_BGH_Rv
5 Mutation/+M162T	1	M162T	pEZZ18_Fw2533	Human_Chia_Rv820
	2	M-9	Human_Chia_Fw735	Sal_BGH_Rv
	Final	5 Mutation/+M162T Part 1 and 2	pEZZ18_Fw2533	Sal_BGH_Rv
5 Mutation/+I173S	1	I173S	pEZZ18_Fw2533	Human_Chia_Rv820
	2	M-9	Human_Chia_Fw735	Sal_BGH_Rv
	Final	5 Mutation/+I173S Part 1 and 2	pEZZ18_Fw2533	Sal_BGH_Rv
5 Mutation/+M179L	1	M179L	pEZZ18_Fw2533	Human_Chia_Rv820
	2	M-9	Human_Chia_Fw735	Sal_BGH_Rv
	Final	5 Mutation/+M179L Part 1 and 2	pEZZ18_Fw2533	Sal_BGH_Rv

5 Mutation/+N190T	1	N190T	pEZZ18_Fw2533	Human_Chia_Rv820
	2	M-9	Human_Chia_Fw735	Sal_BGH_Rv
	Final	5 Mutation/+N190T Part 1 and 2	pEZZ18_Fw2533	Sal_BGH_Rv
5 Mutation/+V246A	1	V246A	pEZZ18_Fw2533	Human_Chia_Rv820
	2	M-9	Human_Chia_Fw735	Sal_BGH_Rv
	Final	5 Mutation/+V246A Part 1 and 2	pEZZ18_Fw2533	Sal_BGH_Rv
5 Mutation/+D252N	1	D252N	pEZZ18_Fw2533	Human_Chia_Rv820
	2	M-9	Human_Chia_Fw735	Sal_BGH_Rv
	Final	5 Mutation/+D252N Part 1 and 2	pEZZ18_Fw2533	Sal_BGH_Rv
5 Mutation/+N270T	1	N270T	pEZZ18_Fw2533	Human_Chia_Rv820
	2	M-9	Human_Chia_Fw735	Sal_BGH_Rv
	Final	5 Mutation/+N270T Part 1 and 2	pEZZ18_Fw2533	Sal_BGH_Rv
5 Mutation/+I272L	1	I272L	pEZZ18_Fw2533	Human_Chia_Rv820
	2	M-9	Human_Chia_Fw735	Sal_BGH_Rv
	Final	5 Mutation/+I272L Part 1 and 2	pEZZ18_Fw2533	Sal_BGH_Rv
5 Mutation/+I300F	1	I300F	pEZZ18_Fw2533	Human_Chia_Rv820
	2	M-9	Human_Chia_Fw735	Sal_BGH_Rv
	Final	5 Mutation/+I300F Part 1 and 2	pEZZ18_Fw2533	Sal_BGH_Rv
M-2/+N270T	1	M-2	pEZZ18_Fw2533	N270T_Rv
	2	M-2	N270T_Fw	Sal_BGH_Rv
	Final	M-2/+T270N Part 1 and 2	pEZZ18_Fw2533	Sal_BGH_Rv
M-2/+T270N,I300F	1	M-2/+N270T	pEZZ18_Fw2533	I300F_Rv
	2	M-2/+N270T	I300F_Fw	Sal_BGH_Rv
	Final	M-2/+T270N,I300F Part 1 and 2	pEZZ18_Fw2533	Sal_BGH_Rv
M-2/+N270T	1	M-2	pEZZ18_Fw2533	N270T_Rv
	2	M-2	N270T_Fw	Sal_BGH_Rv
	Final	M-2/+T270N Part 1 and 2	pEZZ18_Fw2533	Sal_BGH_Rv
M-2/+T270N,I300F	1	M-2/+N270T	pEZZ18_Fw2533	I300F_Rv
	2	M-2/+N270T	I300F_Fw	Sal_BGH_Rv
	Final	M-2/+T270N,I300F Part 1 and 2	pEZZ18_Fw2533	Sal_BGH_Rv
M-2/+M179L	1	M-2	pEZZ18_Fw2533	M-9/-T190N_Rv
	2	M-2	M-9/-T190N_Fw	Sal_BGH_Rv
	Final	M-2/+M179L Part 1 and 2	pEZZ18_Fw2533	Sal_BGH_Rv
	1	M-2	pEZZ18_Fw2533	M-9/-L179M_Rv

M-2/+N190T	2	M-2	M-9/-L179M_Fw	Sal_BGH_Rv
	Final	M-2/+N190T Part 1 and 2	pEZZ18_Fw2533	Sal_BGH_Rv

Supplementary Table S2. Forward and reverse primers for the construction of chimeric and mutant proteins.

Primer name	Sequence
Macaca_Bam_Fw	5’-CGCGGAACCCGGATCCgTACCAGCTGACATGCTACTTCTCCA-3’
Macaca_ XhoI_Rv	5’-GTGACCTCGAGCCCAGCTGCAGCAGGAGCAGGAGGCT-3’
Hum_Bam_Fw	5’-CGCGGAACCCGGATCCgTACCAGCTGACATGCTACTTC-3’
Hum_XhoI_Rv	5’-GTGACCTCGAGCTGGCCAGTTGCAGCAATTACAGC-3’
pEZZ18_Fw2533	5’-AAATGCTGCGCAACACGATGAAGCC-3’
Sal-BGH-Rv	5’-AGGGGTCGACTAGAAGGCACAGTCGAGGCTGATCA-3’
C1_Fw	5’-TGGGCCTGCAGAGTACAAGTTGCACGGCTCCAGCTCAGCC-3’
C1_Rv	5’-GGCTGAGCTGGAGCCGTGCAACTTGTACTCTGCAGGCCCA-3’
C2/C17/C18_Fw	5’-GTAACGCCTACCTCAATGTGGATTATGTCATGAACTACTG-3’
C2/C17/C18_Rv	5’-CAGTAGTTCATGACATAATCCACATTGAGGTAGGCGTTAC-3’
C3_Fw	5’-TCTTCACTGTCCTGGTGCAGGAAATGCGTGAAGCTTTTGA-3’
C3_Rv	5’-TCAAAAGCTTCACGCATTTCCTGCACCAGGACAGTGAAGA-3’
C4_Fw	5’-TCGGCCTGCAGAGTGCAAGTTGCAAAGCTCCAGCCCAACC-3’
C4_Rv	5’-GGTTGGGCTGGAGCTTTGCAACTTGCACTCTGCAGGCCGA-3’
C5/C14/C19_Fw	5’-GCAACGCCTACCTCAATGTGGATTATGCCATCAACTACTG-3’
C5/C14/C19_Rv	5’-CAGTAGTTGATGGCATAATCCACATTGAGGTAGGCGTTGC-3’
C6/C13/C15_Fw	5’-TCTTCACTGTCCTGGTGCAGGAAACGCGTGAAGCTTTTGA-3’
C6/C13/C15_Rv	5’-TCAAAAGCTTCACGCGTTTCCTGCACCAGGACAGTGAAGA-3’
C7/C12_Fw	5’-CCCCCAACTGTCGCAGTACCTGGACTACATCCATGTCATG-3’
C7/C12_Rv	5’-ATGTAGTCCAGGTACTGCGACAGTTGGGGGATCTCGTAGC-3’
C8/C20_Fw	5’-CCCCCAACTGTCACAGTACCTGGACTACATCCATGTCATG-3’
C8/C20_Rv	5’-GATGTAGTCCAGGTACTGTGACAGTTGGGGGATCTCATAG-3’
C9/C16_Fw	5’-GGCCTACTATGAGATCTGTACCTTCCTGAAAAATGGAGCC-3’
C9/C16_Rv	5’-TTCAGGAAGGTACAGATCTCATAGTAGGCCCAGAACCCAG-3’
C10/C21_Fw1	5’-GGGCTTACTACGAGATCTGTACCTTCCTGAAGAATGGAGC-3’
C10_Fw2	5’-TTCCAAATCAAGGCTCAATGGCTTAAGCACAACAAATTTG-3’
C10/C21_Rv1	5’-TTCAGGAAGGTACAGATCTCGTAGTAAGCCCAGATCCCAG-3’
C10_Rv2	5’-TAAGCCATTGAGCCTTGATTTGGAAACTCTTGGTGTTATC-3’
C11_Fw	5’-CTTCGATATTAAGGCTGACTGGCTAAAGAAGAACAACTTC-3’
C11_Rv	5’-TTAGCCAGTCAGCCTTAATATCGAAGCTCTTGATGTTGTC-3’
M-17-1/2/3/8_Ex6_Fw	5’-GCAACAAGCCCAGGCTGCTGGTCACTGCTGCAGTAGCTGCTGGCATCTCCACCATCCAGTCTGGCTATGAG-3’
M-17-1/2/3/8_Ex6_Rv	5’-GCAGCCTGGGCTTGTTGCTCTGCTTGGCCTCCTGCTCAAAAGCTTCACGCGTTTCCTGCACCAGGACAGTG-3’
M-17-1/4/5/6/7/8_Ex8_Fw	5’-GGATTATGCCATCAACTACTGGAAGAACAATGGAGCACCAGCTGAGAAGCTCATCGTTGG-3’
M-17-1/4/5/6/7/8_Ex8_Rv	5’-CCAACGATGAGCTTCTCAGCTGGTGCTCCATTGTTCTTCCAGTAGTTGATGGCATAATCCACATTGAGGTAGGC-3’
M-17-2/4/5_Ex8_Fw	5’-GCTGAGAAGCTCATCGTTGGATTCCCTGCCTATGGACACACCTTCCTTCTGAGCAACCCCTCCGATCATGGAATTGGTGCCCCCACC-3’
M-17-2_Ex8_Rv	5’-GGTGGGGGCACCAATTCCATGATCGGAGGGGTTGCTCAGAAGGAAGGTGTGTCCATAGGCAGGGAATCCAACGATGAGC-3’
M-17-3/4/5_Ex8_Fw	5’-TGGAATTGGTGCCCCCACCACTGGTCCTGGTCCTGCTGGGCCCTATACCAGGGAGTCTGGGTTCTGGGCTTACTACGAGATC-3’
M-17-3_Ex8_Rv	5’-ACCCAGACTCCCTGGTATAGGGCCCAGCAGGACCAGGACCAGTGGTGGGGGCACCAATTCCA-3’
M-17-4_Ex6_Fw	5’-CTTTTGAGCAGGAGGCCAAGCAGAGCAACAAGCCCAGGCTGATGG-3’
M-17-4_Ex6_Rv	5’-CTTGGCCTCCTGCTCAAAAGCTTCACGCGTTTCCTGCACCAGGACAG-3’

M-17-5/6_Ex6_Fw	5'-GCTGCAGTAGCTGCTGGCATCTCCACCATCCAGTCTGGCTATGAGATCC-3'
M-17-5_Ex6_Rv	5'-GATGCCAGCAGCTACTGCAGCAGTGACCAGCAGCCTGGGCTTGTTGATC-3'
M-17-6_Ex6_Rv	5'-CATAGCCAGACTGGATGGTGGAGATGCCAGCAGCTAC-3'
M-17-7_Ex6_Fw	5'-GCAACAAGCCCAGGCTGATGGTCACTGCTGCAGTAGCTGCTGGCATCTCCACCATCCAGTCTGGCTATGAG-3'
M-17-7_Ex6_Rv	5'-TCAGCCTGGGCTTGTTGCTCTGCTTGGCCTCCTGCTCAAAAGCTTCACGCGTTTCCTGCACCAGGACAGTG-3'
M-17-8_Ex8_Fw1	5'-TGGAATTGGTGCCCCCACCACTGGTCCTGGTCCTGCTGGGCCCTATGCCAAGGAGTCTGGGTTCTGGGCTTACTACGAGATC-3'
M-17-8_Ex8_Fw2	5'-GCTGAGAAGCTCATCGTTGGATTCCCTACCTATGGACACACCTTCCTTCTGAGCAACCCCTCCAACACTGGAATTGGTGCCCCCACC-3'
M-12-1_Fw	5'-CCTGGTGCAGGAAATGCGTGAAGCTTTTGAGCAGG-3'
M-12-1_Rv	5'-CAAAAGCTTCACGCATTTCCTGCACCAGGACAGTG-3'
M-12-2_Fw	5'-GGAGGCCAAGCAGATCAACAAGCCCAGGCTGCTGG-3'
M-12-2_Rv	5'-AGCCTGGGCTTGTTGATCTGCTTGGCCTCCTGCTC-3'
M-12-3_Fw	5'-CAAGCCCAGGCTGATGGTCACTGCTGCAGTAGCTG-3'
M-12-3_Rv	5'-CTGCAGCAGTGACCATCAGCCTGGGCTTGTTGCTC-3'
M-12-4_Fw	5'-GCTGGCATCTCCAATATCCAGTCTGGCTATGAGATCCCCC-3'
M-12-4_Rv	5'-TAGCCAGACTGGATATTGGAGATGCCAGCAGCTAC-3'
M-12-5_Fw	5'-CAATGTGGATTATGTCATCAACTACTGGAAGAACAATGG-3'
M-12-5_Rv	5'-TTCCAGTAGTTGATGACATAATCCACATTGAGGTAGGCG-3'
M-12-6_Fw	5'-GTGGATTATGCCATGAACTACTGGAAGAACAATGGAGC-3'
M-12-6/M-11-3_Rv	5'-GTTCTTCCAGTAGTTCATGGCATAATCCACATTGAGG-3'
M-12-7_Fw	5'-TCAACTACTGGAAGGACAATGGAGCACCAGCTGAG-3'
M-12-7_Rv	5'-CTGGTGCTCCATTGTCCTTCCAGTAGTTGATGGCATAATC-3'
M-12-8_Fw	5'-ACCTATGGACACAACTTCCTTCTGAGCAACCCCTC-3'
M-12-8_Rv	5'-GTTGCTCAGAAGGAAGTTGTGTCCATAGGTAGGGAATCC-3'
M-12-9_Fw	5'-CCTATGGACACACCTTCATCCTGAGCAACCCCTCCAACAC-3'
M-12-9_Rv	5'-GGAGGGGTTGCTCAGGATGAAGGTGTGTCCATAGGTAGGG-3'
M-12-10_Fw	5'-TTGGTGCCCCCACCTCTGGTCCTGGTCCTGCTGGG-3'
M-12-10_Rv	5'-AGGACCAGGACCAGAGGTGGGGGCACCAATTCCAG-3'
M-12-11_Fw	5'-CCCCACCACTGGTGCTGGTCCTGCTGGGCCCTATG-3'
M-12-11_Rv	5'-CCAGCAGGACCAGCACCAGTGGTGGGGGCACCAATTC-3'
M-12-12/M-11-2_Fw	5'-AAGGAGTCTGGGATCTGGGCTTACTACGAGATCTG-3'
M-12-12/M-11-2_Rv	5'-GTAGTAAGCCCAGATCCCAGACTCCTTGGCATAGG-3'
M-11-1/6_Fw	5'-GGTGCCCCCACCTCTGGTGCTGGTCCTGCTGGGCCCTATG-3'
M-11-1/6_Rv	5'-GGCCCAGCAGGACCAGCACCAGAGGTGGGGGCACCAATTC-3'
M-11-4/M-9_Fw	5'-TCTGGTGCTGGTCCTGCTGGGCCCTATGCCAAGGAGTCTGGGATCTGGGCTTACTACGAGATCTG-3'
M-11-4/M-9_Rv	5'-TCCCAGACTCCTTGGCATAGGGCCCAGCAGGACCAGCACCAGAGGTGGGGGCACCAATTCCAG-3'
M-11-5_Fw	5'-TCTGGTCCTGGTCCTGCTGGGCCCTATGCCAAGGAGTCTGGGATCTGGGCTTACTACGAGATCTG-3'
M-11-5_Rv	5'-TCCCAGACTCCTTGGCATAGGGCCCAGCAGGACCAGGACCAGAGGTGGGGGCACCAATTCCAG-3'
M162T_Fw	5'-ACTGTCCTGGTGCAGGAAACGCGTGAAGCTTTTGAGCAG-3'
M162T_Rv	5'-CTGCTCAAAAGCTTCACGCGTTTCCTGCACCAGGACAGT-3'
I173S_Fw	5'-GAGCAGGAGGCCAAGCAGAGCAACAAGCCCAGGCTGATG-3'
I173S_Rv	5'-CATCAGCCTGGGCTTGTTGCTCTGCTTGGCCTCCTGCTC-3'
M179L_Fw	5'-GATCAACAAGCCCAGGCTGCTGGTCACTGCTGCAGTAGC-3'
M179L_Rv	5'-GCTACTGCAGCAGTGACCAGCAGCCTGGGCTTGTTGATC-3'
N190T_Fw	5'-GTAGCTGCTGGCATCTCCACCATCCAGTCTGGCTATGAGA-3'

N190T_Rv	5’-TCTCATAGCCAGACTGGATGGTGGAGATGCCAGCAGCTAC-3’
V246A_Fw	5’-TACCTCAATGTGGATTATGCCATGAACTACTGGAAGGAC-3’
V246A_Rv	5’-GTCCTTCCAGTAGTTCATGGCATAATCCACATTGAGGTA-3’
D252N_Fw	5’-TGTCATGAACTACTGGAAGAACAATGGAGCACCAGCTGA-3’
D252N_Rv	5’-TCAGCTGGTGCTCCATTGTTCTTCCAGTAGTTCATGACA-3’
N270T_Fw	5’-TTCCCTACCTATGGACACACCTTCATCCTGAGCAACCCC-3’
N270T_Rv	5’-GGGGTTGCTCAGGATGAAGGTGTGTCCATAGGTAGGGAA-3’
I272L_Fw	5’-ACCTATGGACACAACTTCCTTCTGAGCAACCCCTCCAAC-3’
I272L_Rv	5’-GTTGGAGGGGTTGCTCAGAAGGAAGTTGTGTCCATAGGT-3’
I300F_Fw	5’-CTATGCCAAGGAGTCTGGGTTCTGGGCTTACTACGAGAT-3’
I300F_Rv	5’-ATCTCGTAGTAAGCCCAGAACCCAGACTCCTTGGCATAG-3’
M-9/-T162M_Fw	5’-ACTGTCCTGGTGCAGGAAATGCGTGAAGCTTTTGAGCAG-3’
M-9/-T162M_Rv	5’-CTGCTCAAAAGCTTCACGCATTTCCTGCACCAGGACAGT-3’
M-9/-I173S_Fw	5’-GAGCAGGAGGCCAAGCAGATCAACAAGCCCAGGCTGCTG-3’
M-9/-I173S_Rv	5’-CAGCAGCCTGGGCTTGTTGATCTGCTTGGCCTCCTGCTC-3’
M-9/-L179M_Fw	5’-GAGCAACAAGCCCAGGCTGATGGTCACTGCTGCAGTAGC-3’
M-9/-L179M_Rv	5’-GCTACTGCAGCAGTGACCATCAGCCTGGGCTTGTTGCTC-3’
M-9/-T190N_Fw	5’-GTAGCTGCTGGCATCTCCAATATCCAGTCTGGCTATGAGA-3’
M-9/-T190N_Rv	5’-TCTCATAGCCAGACTGGATATTGGAGATGCCAGCAGCTAC-3’
M-9/-A246V_Fw	5’-TACCTCAATGTGGATTATGTCATGAACTACTGGAAGAAC-3’
M-9/-A246V_Rv	5’-GTTCTTCCAGTAGTTCATGACATAATCCACATTGAGGTA-3’
M-9/-N252D_Fw	5’-TGCCATGAACTACTGGAAGGACAATGGAGCACCAGCTGA-3’
M-9/-N252D_Rv	5’-TCAGCTGGTGCTCCATTGTCCTTCCAGTAGTTCATGGCA-3’
M-9/-T270N_Fw	5’-TTCCCTACCTATGGACACAACTTCCTTCTGAGCAACCCC-3’
M-9/-T270N_Rv	5’-GGGGTTGCTCAGAAGGAAGTTGTGTCCATAGGTAGGGAA-3’
M-9/-L272I_Fw	5’-ACCTATGGACACACCTTCATCCTGAGCAACCCCTCCAAC-3’
M-9/-L272I_Rv	5’-GTTGGAGGGGTTGCTCAGGATGAAGGTGTGTCCATAGGT-3’
M-9/-F300I_Fw	5’-CTATGCCAAGGAGTCTGGGATCTGGGCTTACTACGAGAT-3’
M-9/-F300I_Rv	5’-ATCTCGTAGTAAGCCCAGATCCCAGACTCCTTGGCATAG-3’
Human_Chia_Fw735	5’-CTACGACCTCCATGGCTCCTGGGAG-3’
Human_Chia_Rv820	5’-GGCGTTGCTGCCGGTGTCAGTCGGG-3’
